# Development and Characterization of New Miconazole-Based Microemulsions for Buccal Delivery by Implementing a Full Factorial Design Modeling

**DOI:** 10.3390/pharmaceutics16020271

**Published:** 2024-02-14

**Authors:** Marina-Theodora Talianu, Cristina-Elena Dinu-Pîrvu, Mihaela Violeta Ghica, Valentina Anuţa, Răzvan Mihai Prisada, Lăcrămioara Popa

**Affiliations:** 1 Department of Physical and Colloidal Chemistry, Faculty of Pharmacy, “Carol Davila” University of Medicine and Pharmacy, 6 Traian Vuia Str., 020956 Bucharest, Romania; marina-theodora.talianu@drd.umfcd.ro (M.-T.T.); cristina.dinu@umfcd.ro (C.-E.D.-P.); valentina.anuta@umfcd.ro (V.A.); razvan.prisada@umfcd.ro (R.M.P.); lacramioara.popa@umfcd.ro (L.P.); 2 Innovative Therapeutic Structures R&D Center (InnoTher), “Carol Davila’’ University of Medicine and Pharmacy, 6 Traian Vuia Str., 020956 Bucharest, Romania

**Keywords:** miconazole base, solubilization, UV-spectroscopy, phase diagrams, stabilizers, microemulsions, in vitro drug release, factorial design, critical quality attributes

## Abstract

This research aimed to develop miconazole-based microemulsions using oleic acid as a natural lipophilic phase and a stabilizer mixture comprising Tween 20 and PEG 400 to solubilize miconazole as an antifungal agent known for its activity in oral candidiasis and to improve its bioavailability. The formulation and preparation process was combined with a mathematical approach using a 2^3^-full factorial plan. Fluid and gel-like microemulsions were obtained and analyzed considering pH, conductivity, and refractive index, followed by extensive analyses focused on droplet size, zeta potential, rheological behavior, and goniometry. In vitro release tests were performed to assess their biopharmaceutical characteristics. Independent variables coded X_1_-Oleic acid (%, *w*/*w*), X_2_-Tween 20 (%, *w*/*w*), and X_3_-PEG 400 (%, *w*/*w*) were analyzed in relationship with three main outputs like mean droplet size, work of adhesion, and diffusion coefficient by combining statistical tools with response surface methodology. The microemulsion containing miconazole base–2%, oleic acid–5%, Tween 20–40%, PEG 400–20%, and water–33% exhibited a mean droplet size of 119.6 nm, a work of adhesion of 71.98 mN/m, a diffusion coefficient of 2.11·10^−5^ cm^2^/s, and together with remarked attributes of two gel-like systems formulated with higher oil concentrations, modeled the final optimization step of microemulsions as potential systems for buccal delivery.

## 1. Introduction

Oral candidiasis represents a complex pathology involving imbalances of the oral microbiota, governed by the presence of *Candida albicans* as the main opportunistic pathogen settled in immunocompromised patients [1]. Multiple factors trigger the pathogenic mechanisms of *C. albicans* division [2,3,4,5], setting off local inflammatory processes, epithelial dysfunction, and the appearance of mild to severe oral thrush [6]. The development of pseudomembranous and erythematous plaques induces local pain, sensitivity, burning sensation, and eating dysfunction, compromising the health status of patients [1,7,8].

It is known that buccal tissue is an attractive drug delivery site in both local and systemic targets, being appreciated for some advantages such as the presence of the non-keratinized vascularized epithelium [9,10], ease of administration [11], the avoidance of the hepatic first-pass effect [10], and being tolerated by patients with dysphagia and pediatric groups [12]. Topical treatment in oral diseases focuses on the active ingredient and its maintenance in active concentration at the level of the buccal mucosa [13,14].

Over time, it was proven a safe handle and incorporation of active pharmaceutical ingredients (API) for the treatment of oral candidiasis in various buccal dosage forms like suspensions [15], medical mouthwashes [16,17], gels and in situ forming gels [18,19], varnishes and troches [20], mucoadhesive tablets [21], and versatile hydrogels and polymeric films [22,23,24]. Furthermore, novel nanotherapeutic systems proposed progress in increasing API bioavailability and therapeutic efficacy [25]. Vesicular systems [26], nanoparticles [27], and micro-/nanoemulsified systems [28,29,30] are remarked as the main advances meant to improve the solubility and retention of the antifungals in oral mucosa [6].

Miconazole, an azole-based model drug with clinical efficacy in the treatment of oral candidiasis, acts by inhibition of 14-α-demethylase (CYP51) implied in the ergosterol biosynthesis and alter the integrity of the fungi cell membrane [31]. Additional positive effects repurpose miconazole as an anti-inflammatory agent in skin disorders [32] and a promising cytotoxic via molecular antitumor pathways in particular forms of cancer [33,34]. Miconazole, belonging to the BCS II class, underwent extensive research to improve solubility and sustain an efficient release, thereby enhancing therapeutic efficacy [35,36]. To this debate, Table 1 briefly presents a concise overview of the development of miconazole-based systems designed to improve mucoadhesion and achieve a controlled release. In the most recent findings, polymeric materials can provide excellent support for nanosized-based system inclusion, as seen in the case of nanogels [37,38] and hydrogels loaded with nanoemulsifyable systems [39].

Hosny K. M. et al. defined complex polymeric structures for delivering miconazole with the aid of nanoemulsions, which can be generated in situ after inclusion in a polymeric base [39]. Nanoemulsions are nanocolloids known to provide good solubilization ability for APIs but they are characterized as thermodynamically unstable systems, with drop diameters ranging up to 500 nm [40,41]. The use of small amounts of surfactants (<10%) interferes with the stabilization process and affects the systems’ thermodynamic properties. High-energy methods and a meticulous selection of the cosolvents are required to obtain stable formulations with low droplet size [42].

Microemulsions are versatile nanocolloidal carriers able to sustain drug solubilization and release at the level of buccal mucosa, as it was reported for several natural and synthetic antifungal agents like clove oil in the form of spray-able liquid formulations [28], clotrimazole [43], and itraconazole [30], designed as mucoadhesive structures.

The formation of thermodynamically stable and isotropic systems with droplets with small diameters up to 100–200 nm by the use of optimized levels of surfactants and cosurfactants is considered affordable in designing new platforms for buccal delivery as it was demonstrated for actives from different pharmacological groups like carvedilol [44], prednisolone [45], or triamcinolone acetonide [46]. The common achievements followed in designing microemulsions for buccal delivery are based on improved solubilization and drug release by delivering the medicine in a nano-sized form.

Recent studies revealed new insights into designing polymer-free microemulsions at particular concentration domains of oil, surfactant, cosurfactant, and water [47]. The concept was well researched for itraconazole being included in self-microemulsifying systems made of polyoxyl 35-castor oils, a mixture of triglycerides, and water. The gel-like microemulsions assured a sustained release of the drug in 360 min [48]. The formation of gel-like structures was observed by building phase diagrams and explained by the oil–oil droplet interactions. Moreover, the ability of surfactants to create lamellar structures was linked to the powerful hydration effects established between hydrophilic chains, observed in the case of some microemulsions with monolaurin [49] and gel-like microemulsions for topical delivery of vitamins C and E [47].To understand how the formulation factors impact the critical quality attributes of the microemulsions, a full factorial design can be used to initially screen and process the main effects but also the interactions between input factors [50,51,52,53], creating a promising approach in combining concepts in colloid formulation with mathematical analysis to characterize new architectures with improved therapeutic action.

**Table 1:** Timeline evolution in developing essential miconazole-based formulations designed for buccal application in oral candidiasis.

Year	Pharmaceutical Formulation	API Content	Excipients	Observation	Ref.
1992	Bioadhesive slow-release buccal tablet	10 mg	Modified starch, Carbopol 934 Sodium benzoate, SiO_2_	The tablet formulation exhibited a pronounced antifungal effect at a lower dose compared to commercial gel.	[54]
2003	Mucoadhesive buccal patches	2%	^1^ SCMC, Chitosan, ^2^ PVA, ^3^ HEC, ^4^ HPMC ± ^5^ PVP (0–5%)	Patches had more accurate dosing than the gel form, and the formulation based on PVA 10% and PVP 5% had the best ^6^ MIC release.	[55]
2017	Buccal mucoadhesive films based on polyelectrolyte complexes	2%	Chitosan combined with pectin or HPMC	HPMC increased adhesion and improved the mechanical properties of the chitosan-based films. Improved drug release.	[56]
2017	Buccal mucoadhesive films based on polyelectrolyte complexes	2%	Chitosan + Carbopol, Arabic gum, gelatin, or alginate; ^7^ PEG 400 (30%) used as solubilizer and plasticizer	Chitosan increases antifungal activity of miconazole. Gelatin and Carbopol were appropriate polymers to form chitosan films.	[57]
2017	Mucoadhesive lipid nanogels	0.25–1%	Phospholipon 90 H, Polysorbate 80, beeswax, Polycarbophil, sorbitol	Nanogels with SLN improved the antifungal activity of MIC compared to commercial gel.	[37]
2018	Composite microparticle-based discs	200 mg	Chitosan, gelatin and HPMC	The combination of chitosan-gelatin protects MIC and determines a controlled release. Therapeutic activity can be better improved.	[35]
2018	Buccal films	8%	Chitosan and three types of carrageenan (κ, λ, ι), and PEG 400 as a solubilizer and plasticizer	λ-carrageenan (λ-c) was suitable combined with chitosan. The orientation of sulfate groups in λ-c influenced the interactions with chitosan, but also those with mucin and salivary medium.	[23]
2019	Hydrogels loaded with self-nanoemulsifying drug delivery systems	250 mg	^8^ HA 2%, crosslinked with Gantrez S-97 0.5% which was treated with a NE containing MIC, and clove oil 10–25%, Labrasol 18–70%, and ^9^ PG 10–30%	Labrasol and PG, used as surfactants and cosurfactants in nanoemulsion preparation, determined a high residence of MIC at the mucosal area, enhancing drug permeation. Hydrogel-loaded NE enhanced miconazole release and its contact with the oral mucosa.	[39]
2022	Oral gels	2%	Carbopol 940 and sodium hydroxide, glycerol as a plasticizer, and adjuvants	The optimal gel contained Carbopol 0.84% and sodium hydroxide 0.32%. Miconazole can be prepared in a gel base, influencing texture, spreadability, viscosity and adequate antifungal activity.	[18]
2023	Mucoadhesive nanoparticulate lipospheres	0.25%, 0.5%, 1%	*Bos indicus* fat, Phospholipon 90 H, Tween 80, sorbitol, Polycarbophil	The gel base sustained the delivery of the nanoparticles to the oral mucosa. The nanoparticles’ high surface area increased the contact with the mucosa, while the hydrogel matrix improved mucoadhesion and controlled release.	[38]

^1^ SCMC represents sodium carboxy methyl cellulose; ^2^ PVA—polyvinyl alcohol; ^3^ HEC—hydroxyethyl cellulose; ^4^ HPMC—hydroxy propyl methyl cellulose; ^5^ PVP—polyvinyl pyrrolidone; ^6^ MIC—miconazole; ^7^ PEG 400—Polyethylene glycol 400; ^8^ HA—hyaluronic acid; ^9^ PG—propylene glycol.

To the best of our knowledge, no literature reports are available to consider the formulation of microemulsions with miconazole for buccal applications. Thus, the objectives of the study relied on the development of miconazole-based microemulsions using two levels, three factors full factorial design. The analyses performed in this study focused on increasing miconazole solubility using microemulsions. The systems were preliminarily characterized by considering the analysis of pH, conductivity, and refractive index. In a further stage, the study of droplet size distribution, zeta potential, rheological behavior, superficial properties, and drug kinetic release aimed to explain the internal behavior of microemulsions and their biopharmaceutical performance as potential drug delivery systems. To succeed in the mathematical modeling, three critical quality attributes (CQA, as defined in the Quality by Design QbD approach) (drop diameter, work of adhesion, and diffusion coefficient) were studied by applying response surface methodology. The mathematical analysis was valuable in optimizing a model system that can be tailored in further studies as a biocompatible topical system with mucoadhesive properties.

## 2. Materials and Methods

### 2.1. Materials

The actives and excipients used in the study were of analytical grade. The miconazole base was purchased from Fagron (Rotterdam, Holland). Isopropyl myristate was purchased from Merck Schuchardt (Merck, Hohembrunn, Germany), Oleic acid vegetable from Merck (Merck KgaH, Darmstadt, Germany), Tween 20 from Sigma-Aldrich (Sigma Aldrich Chimie, L’ lsle D’Abeau Chesnes, France) and Tween 80 was purchased from Carl Roth GmbH + CoKG (Karlsruhe, Germany), Kolliphor P407 was acquired from Sigma (Sigma Aldrich, St. Louis, MO, USA) and propylene glycol was supplied from Sigma Aldrich (Steinheim, Germany). Polyethylene glycol 200 and polyethylene glycol 400 were acquired from Scharlau (Scharlab S.L., Sentmenat, Spain). Di-sodium hydrogen phosphate heptahydrate (Merck, Darmstadt, Germany), Potassium dihydrogen phosphate, and ethanol (Chemical Company, Iași, Romania) were selected to prepare the in vitro release medium. Ultrapure Milli-Q water with a specific resistance of 18.2 MΩ/cm and total organic carbon (TOC) of less than 5 μg/L was generated from a Milly-Q^®^ Direct 8 Water Purification System (Merck Millipore, Bedford, MA, USA), and used as the aqueous phase.

### 2.2. Solubility Studies for Miconazole

The miconazole base solubility was tested in eight excipients using the shake flask method [58,59]. The calibration curve of miconazole was projected by preparing standard methanolic solutions of known concentration, as was previously reported [39]. The solutions were prepared by dilution, beginning with a methanolic solution of miconazole of 800 ppm. Each solution was spectrophotometrically measured in UV at λ_max_ = 272 nm, as a function of methanol as the blanc solution, by using Perkin Elmer Lambda 2 UV-VIS spectrophotometer (PerkinElmer Inc., Waltham, MA, USA). The protocol succeeded with the preparation of the miconazole samples of unknown concentration. In 2 mL Eppendorf mini tubes (Eppendorf, Hamburg, Germany), 1 mg miconazole was weighed at the analytical balance (Sartorius MC210P, Sartorius AG, Gottingen, Germany) in 1.5 mL excipient. Miconazole was added until saturation occurred. After weighing each, the tubes were shaken for 5 min at 3500 rotations per minute (rpm). After saturation, the samples were shaken for 24 h at 1000 rpm, at 25 ± 0.5 °C, using the Eppendorf ThermoMixer C (Eppendorf, Hamburg, Germany). After each cycle, the samples were kept for equilibration and centrifuged for 5 min at 15,000 rpm, at 25 ± 0.5 °C, using the centrifuge Micro 200 (Hettich North America, MA, USA). The supernatant was collected and adequately prepared in volumetric flasks using methanol. The samples were spectrophotometrically measured in triplicate at λ_max_ = 272 nm to calculate the solubility.

### 2.3. Screening Study to Design Pseudo-Ternary Phase Diagrams

To explore the stability area for micro-/nanoemulsions formation, four pseudo-ternary phase diagrams were built by applying the water titration method under continuous stirring [60]. Based on the solubility data, oleic acid, a mixture of Tween 20/PEG 400 in four ratios of 2:1, 3:1, 3:2, and 4:1, and water were considered as the three main factors implied in the definition of various coarse dispersions, of which several targeted concentration domains were fixed to build up the factorial plan. Furthermore, for each ratio of S/CoS mix, several ratios of Oil: S/CoS mix (1:9, 2:8, 3:7, 4:6, 5:5, 6:4, 7:3, 8:2, 9:1) were selected to cover a large domain of points and inspect the transitions from stable through unstable formulations. The systems were visualized as transparent or translucent to opalescent systems in the case of micro-/nanoemulsion domain, opaque systems defined as emulsions, particular lamellar phases with a gel-like appearance in the area where the water and the oil phase equally contributed to the generation of dispersions, and unstable dispersions, where creaming and phase separation acted as unfavorable phenomena, as previously found [61]. The plotting was performed using Triplot software, version 4.1.2 (Todd Thompson Software, LA, USA), as reported [62].

### 2.4. Preparation of the O/W Miconazole-Based Microemulsions Using a 2^3^ Full Factorial Plan

The microemulsions with miconazole were obtained under formulation data processing using Design Expert statistical software, version 13. The chosen model to acquire potential microemulsions was a two-level, three-factor full factorial design augmented with two lack of fit points. Firstly, miconazole was accurately weighed at the analytical balance and solubilized in a calculated amount of oleic acid. The lipophilic mixture was placed on a thermostated stirrer (DLAB MS-H380Pro, DLAB Scientific, Beijing, China) for 10 min to attain a clear oily phase. Tween 20 was further weighed, mixed with the lipophilic phase, and subjected to stirring for another 10 min. PEG 400 was weighed and added in the next step to obtain a homogeneous and clear composition. A water titration method with distilled water was applied, and ten microemulsions with various aspects were obtained and placed in equilibration at room temperature. Various structures of microemulsions resulted, from fluid-type to gel-like microemulsions. The fluid microemulsions were prepared using magnetic stirring. For the last case, the homogenization of the samples was assured by trituration during the water titration procedure.

The compositions attributed to the microemulsions designed through mathematical modeling were coded as ME 1-ME 10 and are presented in Table 2. Final compositions of 20 g (%, *w*/*w*) were generated based on the presence of three formulation factors noted X_1_, X_2_, and X_3_.

**Figure 1:**
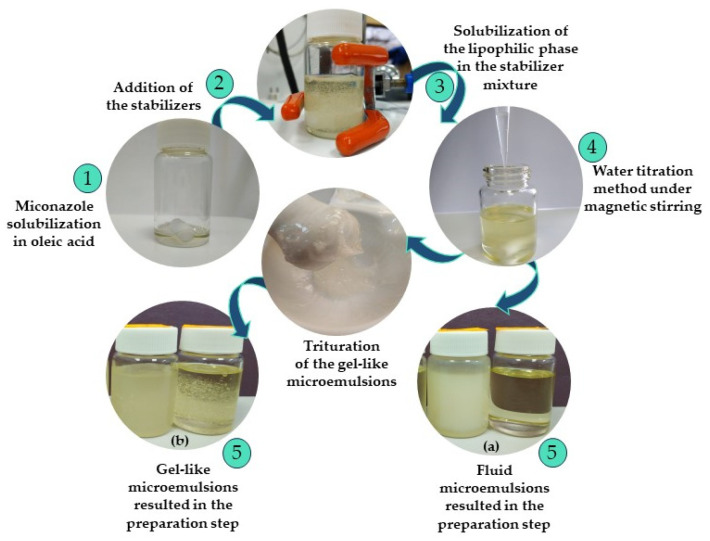
Essential steps followed in the preparation process of the microemulsions with miconazole, consisting in 1— drug solubilization in the oil phase, 2—addition of the stabilizers, 3—lipophilic mixture solubilization with the stabilizer phase under continuous stirring, 4—application of water titration under stirring resulting in two types of systems: 5 (a)—fluid microemulsions, and 5 (b) —gel-like microemulsions, previously triturated to uniformize their consistency.

Figure 1 presents the main steps involved in the preparation process of the fluid and gel-like microemulsions.

### 2.5. Organoleptic Analysis

The microemulsions were visually observed, and their aspect, color, odor, and the presence or absence of instability phenomena were described based on their composition.

### 2.6. pH Determination

The pH of microemulsions was determined using a Mettler–Toledo SevenCompact pH meter (Mettler–Toledo GmbH, Greifensee, Switzerland). A silver-based pH glass electrode that can be used for fluid and gel-like dispersions was connected to the apparatus. Before each measurement, calibration was performed using buffer solutions of pH 4 and pH 7, and then the pH was determined in distilled water [63]. The measurements were recorded in triplicate at 24 ± 0.5 °C.

### 2.7. Conductivity Determination

Conductivity determinations were performed to describe the type of microemulsions and the phase behavior for each sample at 24 ± 0.5 °C. A Corning 441 bench conductivity meter (Cole Parmer Instrument Company, LLC, Vernon Hills, IL, USA) was used, and the measurements were recorded in triplicate.

### 2.8. Refractive Index Determination

The refractive index was studied to inspect the isotropic nature of the samples. The analysis was made using a Krüss DR 201-95 digital refractometer (Krüss Optronic GmbH, Hamburg, Germany). Distilled water with a refractive index of 1.3330 was used as a reference standard for calibration before each test. The measurements were recorded in triplicate at 24 ± 0.5 °C.

### 2.9. Dynamic Light Scattering Determination

Droplet size distribution and polydispersity index (PDI) were studied at 25 ± 0.5 °C, applying a dynamic light scattering (DLS) technique on diluted microemulsions on a ratio of 1:100. Accurate measurements were performed in triplicate using a VascoKin particle analyzer (Cordouan Technologies, Pessac, France), equipped with a 638-nanometer laser, as it was previously reported [63,64]. The estimation of mean droplet diameter was performed considering the presence of spherical particles dispersed in a Newtonian fluid by applying the Stokes–Einstein equation, as can be seen in Equation (1):

(1) $${\mathrm{dH}}_{\mathrm{app}}{=k}_{B}{T/3\pi \eta D}_{\mathrm{app}},$$

where dH_app_ represents the hydrodynamic diameter, k_B_—Boltzmann constant, T—the absolute temperature, η—the viscosity of the medium, and D_app_—the apparent diffusion coefficient estimated from the autocorrelation function.

The droplet size profiles projected using the Cumulant model were visualized comparatively to find homogeneous systems with a narrower domain of droplet dimensions, with peaks displaced through a 100 nm size zone.

### 2.10. Zeta Potential Analysis

The Doppler Laser electrophoresis principle was applied to study the droplet charge of the microemulsions. The Wallis Zeta potential analyzer (Cordouan Technologies, Pessac, France) uses a 20 mW diode laser source with a wavelength of 635 nm [65]. A 50% aqueous suspension of colloidal silica—Ludox TM-50 (Sigma Aldrich, St. Louis, MO, USA), diluted in a ratio of 1:100, with a zeta potential of 40 mV was used as a reference standard. The results fitted with the Smoluchowski model were presented as the mean values of ten consecutive determinations for each tested sample.

### 2.11. Rheological Evaluation

The flow behavior of the microemulsions was tested at 37 ± 0.5 °C using a Lamy RM100 CP2000 Plus rheometer equipped with a cone-plate stage (Lamy Rheology Instruments, Champagne au Mont d’Or, France) [66]. The cone-plate coded CP6020 was used, and the measurements were performed by applying 17 rotational speeds from 0.3 rpm to 60 rpm. Shear rate (s^−1^), shear stress (Pa), and viscosity (Pa·s) were recorded, and the flow behavior was described using mathematical modeling.

### 2.12. Superficial Analysis

Extensive analyses of the superficial properties of microemulsions and their wettability behavior were based on measuring the free superficial energy and contact angle. CAM 101 Goniometer, equipped with a Hamilton syringe, a C209-30 needle, and a digital camera (KSV Instruments Ltd., Espoo, Finland), was used as previously reported [63]. Drops of each sample (μL) were applied on microscope slides, captured with a digital camera, and measured throughout 64 ms by an automated curve-fitting program. The determinations in the pendant drop model were performed in triplicate, while those in the contact angle model were made in quintuplicate at 24 ± 0.5 °C.

Young–Laplace equation (Equation (2)) was used to automatically analyze the drop shape in the *pendant drop* model, while the Young equation (Equation (3)) was specific for the *contact angle* model:

(2) $$\Delta p=\;p_{\mathrm{int}}{-\;p}_{\mathrm{ext}}=\gamma_{\mathrm{LG}}\left(\frac{1}{r_{1}}+\frac{1}{r_{2}}\right),$$

(3) $$\gamma_{\mathrm{SG}}=\gamma_{\mathrm{SL}}+\gamma_{\mathrm{LG}}\cos\theta ,$$

where Δp represents the pressure difference between internal and external areas of a curved liquid, also known as Laplace pressure; r_1_, r_2_—the principal radii of curvature; γ_SG_—the interfacial tension to solid/gas (S/G) interface; γ_SL_—interfacial tension to solid/liquid (S/L) interface; γ_LG_—superficial tension to liquid/gas (L/G) interface; and θ—the contact angle made by the liquid drop with the solid surface, which gave information concerning the wettability of microemulsions.

To better explain the drop dynamics at the level of a surface, the Young–Dupré equation was further followed to calculate the work of adhesion (W_a_) and work of cohesion (W_c_) [63]. In a deeper understanding, the spreadability coefficient (W_s_) was linked to the parameters mentioned above, according to the Harkins theory of spreading [67]. The equations used in the analysis for the last three parameters are shown below (Equations (4)–(6)):

(4) $$W_{a}{=\gamma }_{\mathrm{LG}}\left(1+\cos\theta \right),$$

(5) $$W_{c}{=2\gamma }_{\mathrm{LG}},$$

(6) $${S=\gamma }_{\mathrm{LG}}\left(\cos\theta -1\right),$$

where W_a_ represents the work of adhesion calculated using the Dupré equation; W_c_—the work of cohesion; S—the work of spreading or spreading coefficient, which derives from Harkins’ theory of spreading; γ_LG_—the superficial tension to L/G interface; and θ—the contact angle determined previously by applying the contact angle model.

### 2.13. In Vitro Release Studies

The in vitro release of miconazole from the designed microemulsions was performed using a system based on six Microette vertical diffusion cells (Teledyne Hanson Research, Hanson, USA) [68]. Hydrophilic membranes of cellulose acetate of 0.45 μm pore diameter and 120 μm thickness (Sartorius Stedim Biotech GmbH, Goettingen, Germany) were chosen according to previous reports [47,69]. The diffusion cells presented an effective diffusional area of 1.77 cm^2^ and 7 mL of receptor cell capacity. Phosphate buffer with a pH of 6.8 containing 20% ethanol (*w*/*w*) was used as a receptor medium, ensuring sink conditions [24]. A magnetic bar was immersed in each receptor compartment, and the stirring was set to 300 rpm. The membranes were wetted in the medium 12 h before the experiment and were adequately placed between donor and receptor compartments of the diffusion cells. Samples of 1 g were placed into each donor compartment using a cell adapter for fluid formulations [70]. The operational temperature in the diffusion cells was maintained at 37 ± 0.5 °C using a thermostated Julabo Corio CD circulating water bath (Julabo GmbH, Seelbach, Germany). An amount of 1 mL of sample from each receptor medium was collected at predetermined times and replenished with an equal volume of fresh medium to maintain a constant volume. The drug content was spectrophotometrically quantified in UV at 272 nm, and the mechanism of drug release was further determined following the Higuchi model (Equation (7)). The release profiles of miconazole from the designed microemulsions were compared to the release of miconazole from a commercial gel (Daktarin 2% oral gel, Esteve, Spain).

(7) $$D_{m}=\frac{q^{2}\pi }{{4\;C}_{0}^{2}t}$$

where D_m_ represents the diffusion coefficient of MCZ in the release medium, q—the amount of MCZ released on the surface unit, C_0_—the initial MCZ concentration in the microemulsion, and t—the release time.

### 2.14. Data Analysis and Screening of the Miconazole Microemulsions Using 2^3^ Full Factorial Design

The screening process of the microemulsions was designed using a regular two-level full factorial design with two levels of variation and three factors (2^3^). The model was generated using Design-Expert software, version 13 (Stat-Ease, Inc. Minneapolis, MN, USA) [62] and was projected as a 2^3^ factorial plan with eight experiments, augmented with a supplementary block of experiments, specifically two lack-of-fit points. In this sense, three independent variables were selected to design microemulsions at two levels of variation, coded as low (−1) and high (+1): X_1_: Oleic acid (%), X_2_: Tween 20 (%), X_3_: PEG 400 (%). The augmentation proposed the introduction of intermediary lack of fit points. Thus, the experimental design consisted of the preparation of 10 formulations equivalent to 10 runs and the analysis of three responses as dependent variables, noted as Y_1_: droplet size (nm), Y_2_: work of adhesion (mN/m), and Y_3_: diffusion coefficient (cm^2^/s).

Each response was associated with a mathematical interpretation through a linear polynomial equation generated by software in the analysis step. To determine the significance of the analyzed model, the independent variables, and their possible interactions, the equations were statistically analyzed using ANOVA, assuming *p* < 0.05. The models were explained by applying response surface methodology based on a graphical interpretation using contour, surface response, and interaction plots. Valuable prototypes with specific characteristics were identified from the results as models to follow in prospective studies centered on buccal delivery, and predictive optimization was considered in the final step.

## 3. Results and Discussion

### 3.1. Solubility Studies

The solubility of miconazole in oils, surfactants, and cosurfactants was established through the spectrophotometric technique and presented in Table 3. The selected excipients have been widely used in the preparation of microemulsions, being selected for solubilizing antifungals for multiple routes of administration [71,72], including buccal delivery [39].

Among the potential two oils selected, the highest solubility was obtained for oleic acid (110 ± 5 mg/mL). The stabilizer component with good solubilization properties was composed of Tween 20 (236 ± 11 mg/mL) and PEG 400 (209 ± 13 mg/mL), which contributed to the formation of microemulsions.

The use of oleic acid in pharmaceutical systems is not limited just to its role as a lipophilic phase [73]. Oleic acid is an unsaturated fatty acid known to increase drug localization into tissues, being a potent penetration and permeation enhancer for even big and small molecules, in combination with various molecules like PEG 200, PEG 400, or poloxamers [74]. In the study of Yang T-L. et al., oleic acid was used to obtain self-microemulsifying systems with clotrimazole. Oleic acid synergistically enhanced the antifungal activity of clotrimazole through particular mechanisms acting against filamentation and biofilm formation [75].

From the group of surfactants, Tween 20 exhibited the highest solubilization ability for miconazole. PEG 400 was selected as a suitable cosurfactant generally recognized as a solubilizer, humectant, and plasticizer in various pharmaceutical formulations. Several contributions proposed the inclusion of PEG 400 in some model nanocolloidal systems for drug solubilization. PEG 400 1–2.5% was included in the form of gel-based nanoemulsions containing 6-gingerol 10%, oleic acid 1–5%, Tween 20 4–10%, and water 82.5–94%, for wound treatment [76]. PEG 400 6.5–10% was selected in formulations designed for intranasal delivery to increase the rate of survival in glioblastoma treatment [77], while concentrations of 15–21% were selected for designing microemulsions for the oral delivery of carvedilol as suitable solubilizer that acts synergistically with peppermint oil 10% and Tween 80 15–21% [78]. Amounts of 6–9% PEG 400 were tested for antifungal buccal sprays, emphasizing the importance of the stabilizers for microemulsion development [28].

### 3.2. Screening Study to Design Pseudo-Ternary Phase Diagrams

Depiction of the stability area of microemulsions is considered an important step to proceed before the preparation process of the microemulsions. It is well known that micro-/nanoemulsions are considered self-emulsified nanodispersions with common properties in a matter of dimensional domain but with a different behavior concerning structural aspects, thermodynamic and kinetic stability [79]. However, confusion persists over the subject, and novel studies have come out to reveal particular aspects of their formation. Firstly, in the so-called paradox of drop dimension, nanoemulsions were studied as nanodispersions with oil drop diameters that attain 20–200 nm [80] up to 500 nm, according to several reports [41]. On the other hand, microemulsions’ droplet size is concentrated in the domain of nanoemulsions, restricting up to 100–200 nm, due to the presence of high concentrations of S/CoS mix reaching up to 70% of the total composition. The high thermodynamic stability and clear appearance of microemulsions promoted by the self-emulsification technique continue to draw attention in the area of drug delivery and beyond [81,82].

**Figure 2:**
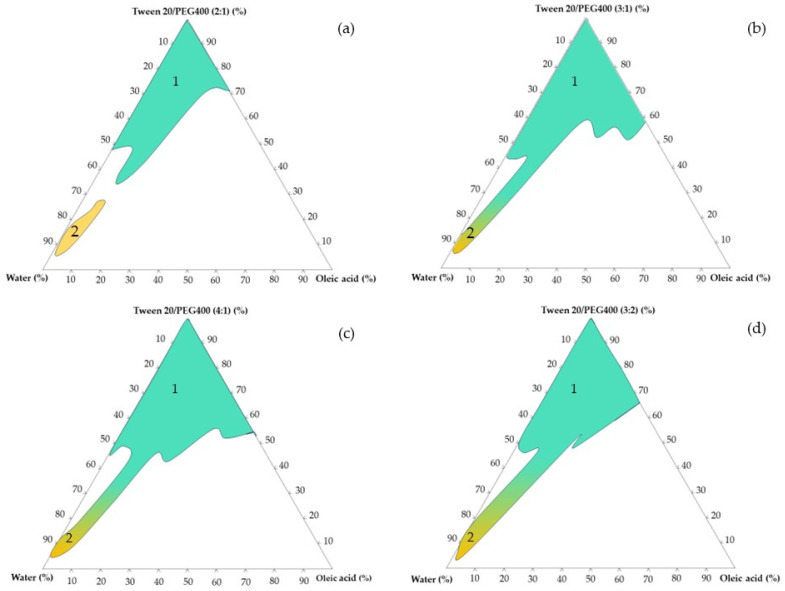
Pseudo-ternary phase diagrams for coarse dispersions containing a stabilizer mixture based on (**a**) Tween 20/PEG 400 in a ratio of 1:1, (**b**) Tween 20/PEG 400 in a ratio of 2:1, (**c**) Tween 20/PEG 400 in a ratio of 3:1, and (**d**) where 1 represents the area of microemulsions (in green color) and 2is the area of nanoemulsions (in yellow color).

The initial step of pseudo-ternary diagram plotting offered an indicative perspective concerning the area in which micro-/nanoemulsions could be generated. An essential goal of the experiment consisted of the depiction of instability points and the avoidance of inappropriate preparation trials. This approach was considered helpful in determining the main concentrations of the oil and stabilizers that can be integrated into a factorial design to further succeed in the development of microemulsions with miconazole. In Figure 2 are presented pseudo-ternary phase diagrams for coarse dispersions containing stabilizer mixtures based on (**a**) Tween 20/PEG 400 in a ratio of 2:1, (**b**) Tween 20/PEG 400 in a ratio of 3:1, (**c**) Tween 20/PEG 400 in a ratio of 4:1, and (d) Tween 20/PEG 400 in a ratio of 3:2. The areas specific for each type of dispersion were projected, patterned, and noted as follows: 1 represents the area of microemulsions (in green pattern) and 2 represents the area of nanoemulsions (in yellow pattern) which collides with the microemulsion zone (cases (b), (c) and (d)).

**Figure 3:**
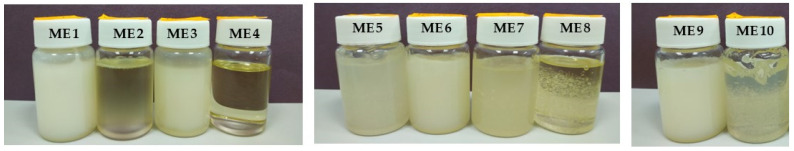
The microemulsions coded ME 1–ME 10 visualized after preparation at room temperature, 24 ± 0.5 °C.

It was concluded that microemulsions could be adequately obtained if the Tween 20/PEG 400 ratio is selected in a ratio of 3:1 or 4:1, as represented in Figure 3—cases (b) and (c). The area of microemulsions was represented with a green color, while the yellow-like area was defined as a particular area of nanoemulsions. As the ratio of Tween 20/PEG 400 is modified through 2:1 and 3:2, the area of stability becomes narrowed, especially in the case of the O/W systems. At the same time, the unstable area was wider in the two last cases. Tween 20 significantly contributed to an enlargement of the microemulsion area. The method was considered useful to appreciate potential domains of stable microemulsions that can be proposed and studied using a factorial model.

### 3.3. Formulation Design and Organoleptic Analysis

The screening design was concentrated on the generation of two levels, three factors full factorial plan. The three variables selected in this design are presented in Table 4. The levels of variation were coded as low (−1) and high (+1). For the model’s reliability, the factorial plan was augmented with two more experiments, and here, two lack of fit points were generated by the software, resulting in a 2^3^ + 2 model.

The full matrix describing the factorial model is presented in Table 5. It is composed of the main block specific for 2^3^ experiments and the second block with the augmented points.

One day after preparation, microemulsions were visually inspected. The aspect, color, and odor were dependent upon composition. The concentration of Tween 20 influenced the aspect, determining the clarity or the opalescent appearance, as can be seen in Figure 3. The systems were predominantly opalescent in the selected lower concentration of Tween 20 30%. An increase of 10% of Tween 20 modified their clarity. ME 2, ME 4, ME 8, and ME 10 were the clearest systems formulated with a concentration of 40% Tween 20, but also 30% in the case of ME 10. On the other side, an increase in concentration for PEG 400 from 10% to 20% improved the aspect, obtaining clear microemulsions only in the presence of Tween 20 40%. Variation of the oil phase from 5% up to 10% influenced the internal structure of the samples, resulting in four systems with gel-like appearance, namely ME 5–ME 8. In the case of the systems from Block 2, ME 9 had a similar composition with the fluid group of MES, while ME 10 was representative of a lack of fit point derived from the gel-like group. The replication (for the last two MES) was automatically obtained, resulting in viscous systems as ME 7. ME 1, ME 3, and ME 9 were the only systems that encountered instability phenomena during the study.

### 3.4. pH Determination

Over the pH analysis, it was observed that the values varied between 5.15 ± 0.02 and 5.80 ± 0.02, being influenced by composition. It can be stated that both the stabilizers and the presence of oleic acid modulated pH variation. Tween 20 and PEG 400 were implied in pH increasing, while the acidic group grafted in the structure of oleic acid determined a decrease in pH, which can be seen in some pairs like ME 1 and ME 5, ME 2 and ME 6, and ME 3 and ME 7. The maximum pH values were obtained for ME 4 and ME 8, characterized by a maximum content of the stabilizers. The normal salivary pH varies between 6.2 and 7.6 [83], while in the pathological state, the pH of the oral cavity changes and tends to be lowered (favorable for bacterial and fungi development) [84]. The pH of ME 4 and ME 8 were closer to 6 and may positively interfere with promoting an antifungal effect in the affected oral mucosa. The results of pH obtained in triplicate for each microemulsion are presented in Table 6.

### 3.5. Conductivity Determination

Conductivity analysis was performed to confirm the O/W type of the microemulsions [85]. The results obtained and presented in Table 6 showed a conductivity variation between 7.92 ± 0.02 and 101.90 ± 0.66 μS/cm, where the maximum value was specific for the microemulsions with the highest water level of 53%. Furthermore, by increasing Tween 20 from 30% to 40% or PEG 400 from 10% to 20%, conductivity decreased to 47.60 ± 0.10 μS/cm and was well visualized for the ME 1–ME 4 group. With the addition of the oil phase in the maximum concentration of 10%, a strong effect can be observed, attaining minimum values around 8 μS/cm, specific for ME 7, its replications, and ME 8. In the case of ME 9 and ME 10, intermediary results were obtained.

### 3.6. Refractive Index Determination

Refractive index (RI) variation between 1.3823 ± 0.0002 and 1.4318 ± 0.0001 was specific for O/W microemulsions with isotropic characteristics [63]. The main ingredients that influenced RI as a quality parameter were oleic acid, Tween 20, and PEG 400. The RI values were shifted after the value of 1.3330, specifically for water as a reference over determinations. The variation in RI values was attributed to a higher refractive index of surfactants and the oil phase [85,86].

The microemulsions characterized by a high clarity and high RI were those prepared with at least one increase of the three ingredients. The first increase can be seen in the pair ME 1–ME 2, where Tween 20 increase from 30% to 40% determined a RI of 1.4047 ± 0.0001. The same phenomenon was observed in the case of the ME 1–ME 3 pair, where only PEG 400 was modified. When Tween 20 and PEG 400 increased simultaneously, RI attained 1.4238 ± 0.0001 for ME 4. The second increase was obtained when oleic acid concentration was fixed at 10%. Together with the modulation in the concentration of the previously mentioned stabilizers, the refractive index variation was similar and attained the maximum value of 1.4318 ± 0.0001 for ME 8. Intermediate values of RI were obtained for ME 9 and ME 10 systems, where the maximum value was attributed to ME 10.

### 3.7. Rheological Evaluation

**Figure 4:**
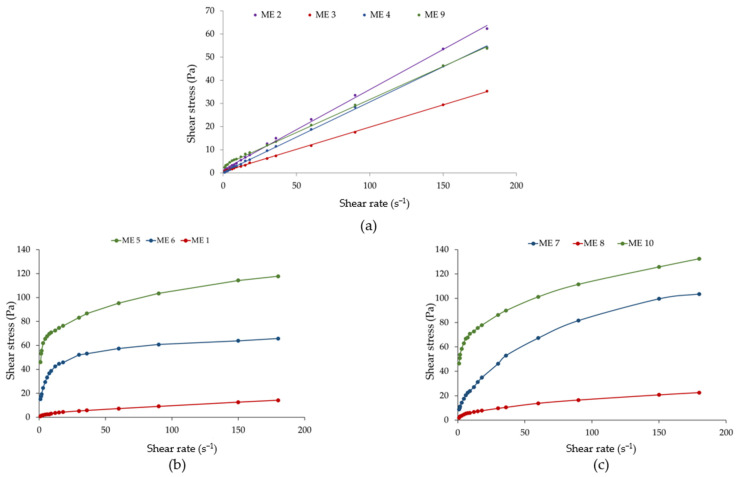
Plots of shear stress (Pa) as a function of shear rate (s^−1^) following specific rheological models: linear profiles for ME 2–ME 4 systems described by the Newton’s model, and ME 9 described by Bingham model—case (**a**); flow curves fitted by the Ostwald–de Waele model for ME 1 and ME 8 systems, and Herschel–Bulkley model specific for ME 5–ME 7 and ME 10 gel-like systems—cases (**b**,**c**).

Following rheological evaluation, two distinctive groups of microemulsions were assessed and described under four rheological models. The fluid-type microemulsions, namely ME 2, ME 3, and ME 4, exhibited a Newtonian flow, with viscosities placed in the narrow domain of 0.192 Pa·s and 0.346 Pa·s. In this case, the increase of Tween 20 and PEG 400 concentration diminished the viscosity. Graphical representations of shear stress (Pa) as a function of shear rate (s^−1^) are presented in Figure 4—case (a). The linear model was mathematically described by Newton’s Law and validated by adequate rheological descriptors, with correlation coefficients between 0.9993 and 0.9998. Newtonian behavior remains a classical model for fluid microemulsions with a laminar flow, keeping a constant viscosity with the increase in the shear stress and shear rate [87,88]. In this case, the rheological parameters assessed from the regression equations are presented in Table 7.

On the other side, the non-Newtonian flow behavior was specific for the microemulsions for which viscosity varied with the shear rate at constant temperature. ME 9 followed an ideal plastic flow described by the Bingham model. The linearity is kept, but the regression straight line was designed with a start point from an initial yield stress (τ_0_) of 3.207 Pa, as presented in Figure 4—case (a). Bingham model was described in a previous study by Djekic, L. et al. for some model microemulsions with ibuprofen at particular composition with isopropyl myristate 5.50%, Labrasol 19.80–29.70%, cosurfactant 19.80–29.70% and water 45% [89]. In a recent report, Bingham-type microemulsions were associated with the formation of flocculated systems characterized by frictional forces as determinants for the initial yield stress recorded [90], being applicable in the case of ME 9, likewise.

In contrast, pseudoplastic behavior is well defined in ME 1 and the gel-like type ME 5, ME 6, ME 7, ME 8, and ME 10. Rheological plots of shear stress (Pa) as a function of shear rate (s^−1^) are represented in Figure 4—case (b) and (c) for the model systems. The Ostwald–de Waele and Herschel–Bulkley models fitted the non-Newtonian pseudoplastic flow with correlation coefficients between 0.9654 and 0.9993. In this case, the graphical representations show ascendent rheograms outlined by rheological descriptors, namely consistency index—K (Pa·s^n^) and flow index values under 1 (n < 1), which were depicted from regression equations, as were presented above in Table 7.

A particular behavior was noticed in this group as a function of the influence of the critical formulation factors upon the internal structure of the microemulsions. Firstly, ME 1 had the lowest consistency index, with a fluid-like appearance, and it was characterized by minimum levels of oil, surfactant, and cosurfactant.

At the increase in the oil phase from 5% to 10%, the microemulsions were characterized by various gel-like structures with shear-thinning behavior. In the case of ME 5, the maximum consistency index was 30.89 Pa·s, obtained at the minimum level of Tween 20 and PEG 400, which signifies the establishment of strong interactions between components. The Herschel–Bulkley model assumed in this case a flow profile starting from τ_0_ of 19.60 Pa. A similar behavior was seen for ME 10, where τ_0_ was equal to 28.17 Pa. When Tween 20 increased to 40%, the consistency coefficient decreased at 21.81 Pa·s^n^ for ME 6, and it continued to diminish by 2.6 folds in the case of ME 7. ME 8 had a lower consistency coefficient of 2.213 Pa·s^n^ at the maximum concentrations of Tween 20 and PEG 400, these excipients being implied in fluidifying the microemulsions. Considering the rheograms presented in Figure 4—cases (b) and (c), as the amount of the stabilizer increased, a transition from the Herschel–Bulkley model to the Ostwald–de Waele flow type was assessed.

The rheological behavior in microemulsions is viewed in the literature in perfect harmony with the internal transitions, which depend on the water and oil content, triggering significant changes in viscosity [91]. Secondly, the nature of the surfactants and cosurfactants modulates the viscosity, as was seen in a previous report centered on the effect of glycols in the microemulsion formation [88]. It was stated that viscosity can be influenced by the drug solubilization in the oil phase. At lower oil concentrations, drug molecules can be solubilized nearest the interface area, determining a lack of droplet interactions and reduced availability to create intermolecular hydrogen bonds [89].

When the oil phase is increased at 10%, the drug molecules are solubilized in a deeper core, determining the creation of organized networks that modify the rheological properties of the systems. Suggestive findings describe the rheological properties of liquid crystals containing oleic acid 10–20%, a mixture of esters of phosphoric acid and the polyoxypropylene, polyoxyethylene ether of cetyl alcohol (PPG-5-CETETH)-20 50%, and water 30–40%, as intermediary systems discovered in the ternary phase diagram. Herschel–Bulkley model was representative of systems with a consistency index of 0.04–1.17 Pa·s^n^, with values that were raised to 3.63–29.34 Pa·s^n^ after adding ursolic acid [92]. The gel-like microstructure of some microemulsions with itraconazole for buccal application was highlighted, resulting in systems with K values between 0.0156–2.895 Pa·s^n^ and flow index values of 0.361–1.1817. In this case, the pseudoplastic behavior was emphasized using the Power Law model [48]. Rozman B. et al. analyzed the viscosity of a gel-like microemulsion prepared with isopropyl myristate 9.86%, Tween 40 14.79%, Imwitor 308 14.79%, and water 59.16% to incorporate vitamin C 0.4% and vitamin E 1%. In this case, at a testing temperature of 20 °C, the pseudoplastic behavior was preserved. In comparison, in the case of a modified temperature of 32 °C, the system suffered structural changes following the Newtonian flow model [47].

Notably, the structural attributes in microemulsions depend upon the composition and govern the flowability and spontaneous changes in the internal behavior. This way, the rheological modeling creates a pathway to assess the strong connections between viscosity and the droplet size distribution impacting the biopharmaceutical properties of the designed microemulsions.

### 3.8. Dynamic Light Scattering Analysis

Dynamic light scattering technique was applied to determine the mean droplet size distribution in miconazole-based microemulsions using the Cumulant algorithm. The samples were diluted with distilled water in a ratio of 1:100, and the resulting dispersions were analyzed at 24 ± 0.5 °C. According to the Rayleigh phenomenon of light dispersion, diffusion coefficients were depicted and then integrated into the Stokes–Einstein equation. The mean hydrodynamic diameter was estimated as Z_average_ and was considered one of the main critical quality attributes included as a response in the statistical analysis and optimization process.

The values of mean droplet size (Ds) obtained in this stage varied between 119.60 ± 1.37 nm and 250 ± 6.50 nm and are presented in Table 6 as means of three different acquisitions. The results are accompanied by polydispersity index (PDI) values, which varied between 0.165 ± 0.003 and 0.301 ± 0.007, being used to appreciate the homogeneity of the systems and the distribution of the droplets. Over the analysis, the data assessed in this analysis present the Ds distribution of microemulsion droplets using the D_10%_, D_50%_, and D_90%_ domains presented in Table 6, being extrapolated to the profiles generated by the software and expressed as intensity (a.u.) as a function of diameter (nm). The Span values were calculated to evaluate the size distribution width. In the case of the microemulsions with miconazole, a limited variation between 1.06 and 1.47 was found. It is unanimously accepted that the droplet size is not part of a single narrowed domain but may detect some extended droplet sizes that can be evaluated from the peak distribution, as was reported. In the study of Monton C. et al., the incorporation of clove oil 2% into microemulsions stabilized with Tween 80 9–18% and PEG 400 6–9% was quantified by sized distributions in a maximum range of D_10%,_ D_50%,_ D_90%_ of 20.7–686.6 nm, with Span values between 1.3 ± 0.0 and 16.3 ± 1.3 [28].

**Figure 5:**
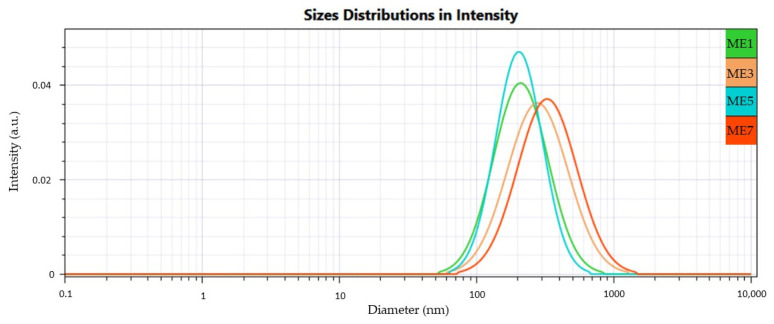
Distribution profiles obtained in the DLS study and represented as intensity (a.u.) as a function of diameter (nm) for ME 1, ME 3, ME 5, and ME 7, evaluated at 24 ± 0.5 °C.

**Figure 6:**
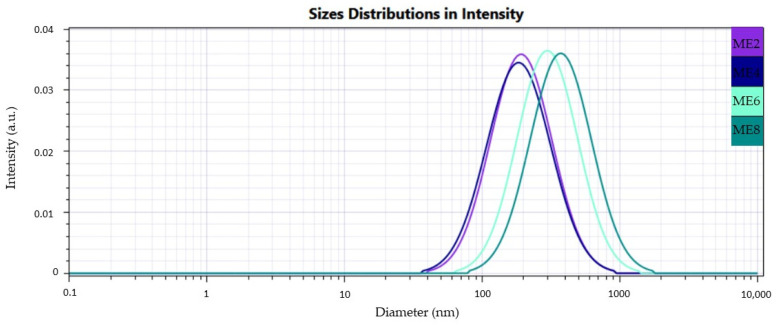
Distribution profiles obtained in the DLS study and represented as intensity (a.u.) as a function of diameter (nm) for ME 2, ME 4, ME 6, and ME 8, evaluated at 24 ± 0.5 °C.

**Figure 7:**
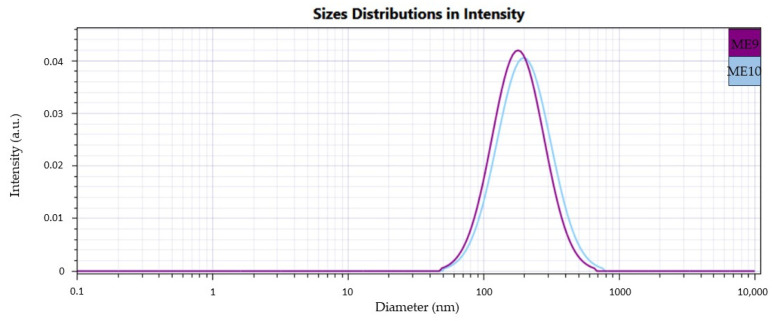
Distribution profiles obtained in the DLS study and represented as intensity (a.u.) as a function of diameter (nm) for ME 9 and ME 10, evaluated at 24 ± 0.5 °C.

Herein, Figure 5, Figure 6 and Figure 7 present cumulative profiles of microemulsion droplet distribution in three groups to observe the peak shifts from 200 nm to 100 nm as a function of their composition.

From a statistical point of view, it was observed that oleic acid and PEG 400 were significant factors implied in droplet size variation. At the same time, the surfactant had a behavior less pronounced and partially understood (*p* < 0.05). By associating the Z_average_ results with the microemulsion compositions, particular dynamics were observed in droplet behavior as a function of the three main formulation factors. As such, at a constant level of oleic acid of 5%, some observations can be made in the ME 1–ME 4 group:

- An increase of Tween 20 content from 30% to 40% determined a decrease in Ds from 152.89 nm to 128.90 nm in the case of the ME 1–ME 2 pair and from 188.33 nm to 119.60 nm in the case of the ME 3–ME4 pair; an additive effect of PEG 400 was observed in the ME 2–ME 4 pair where Ds decreased from 128.90 nm to 119.60 nm.
- At the minimum concentration of Tween 20 of 30%, the increase of PEG 400 determined an increase in droplet size, as can be observed in the case of the ME 1–ME 3 pair, acting oppositely as it was proposed in the mathematical modeling of the Ds response.

In the second group, the presence of oleic acid in the maximum concentration involved an increase in droplet diameter, with a high impact on the broadening of the distribution domain:

- An increase of Tween 20 from 30% to 40% determined an increase of Ds from 161.34 nm to 202.29 nm in the case of ME 5–ME 6 pair, but also from 225.13 nm to 250.20 nm for ME 7–ME 8 pair.
- When Tween 20 is maintained as constant, PEG 400 variation from 10% to 20% promoted growth in droplet size, and it can be very well emphasized in the case of the ME 5–ME 7 pair and ME 6–ME 8 pair, where the maximum droplet size of 250.20 nm was attributed for ME 8.

In the case of the ME 9 and ME 10, intermediate values were obtained.

Considering the above observations, a similar behavior was reported in the literature for PEG 400 chosen in the concentration of 6–9%, which determined an increase in droplet size in microemulsions prepared with clove oil 2% and Tween 80 9–18% as potential oral sprays for buccal application in oral candidiasis [28]. Using its high solubilization ability, Tang H. et al. observed an increase in droplet size of self-microemulsifying drug delivery systems (SMEDDS) with resveratrol when PEG 400 was selected between 5–35%. The maximum concentration of PEG associated with a lower surfactant concentration contributes to the expansion of the interfacial area, affecting the system stability [93]. Moreover, Badawi N.M. et al. reported that in some cases, the surfactant molecules can increase droplet size by forming micellar aggregates that may hinder their normal deposition at the oil/water interface [94]. On the other hand, the microemulsions prepared with oleic acid 10%, but also the ME 10 containing oleic acid 8.75% have a different structure compared to those containing a lower oil concentration. The oil phase promoted an increase in viscosity and generation of gel-like systems with particular interactions between oil, the stabilizers, and water, which will be further relevant for the adhesion properties and the drug release mechanism.

### 3.9. Zeta Potential Analysis

Zeta potential was specific for positive-charged microemulsion droplets, with experimentally recorded values between +9.10 mV and +14.00 mV, as presented in Table 6. The parameter was important in appreciating stability and explaining phenomena implied in droplet stabilization [95]. Zeta potential was studied in various micro-/nanoemulsified systems [96,97], including microemulsions with antifungal agents [28,98]. It was clarified that stabilization can be influenced not only by electrostatic repulsions but also by steric stabilization, promoted by polysorbate-based surfactants [98] and dispersion forces [59]. It was stated that non-ionic surfactants and PEG 400 are not charged contributors, and therefore, zeta potential is influenced by other molecules [99]. In the present study, the electrostatic repulsions between positively charged droplets can be justified by the presence of miconazole base, which at a weakly acidic pH is found in a protonated state [100]. The results can be correlated with recent findings for solid nanoparticles created with stearic acid and Tween 20 to entrap miconazole nitrate. The pH dependence of the systems was proved using indomethacin as an API with an acidic group that modifies zeta potential through negative charging. A negative charge was also recorded for the blank system, where stearic acid behaves as an adsorption component implied in electrostatic stabilization [101]. In the present study, steric stabilization was more pronounced than electrostatic stabilization. Carrying a lower positive charge, the studied microemulsions may interact with negatively charged glycoprotein groups in the oral mucosa, as previously observed for positively charged nanoparticles [102]. However, prospective research must be considered to demonstrate such a mechanism that could be beneficial in providing therapeutic efficacy.

### 3.10. Superficial Analysis

The study of superficial properties in colloid science offers an advanced perspective by understanding fundamental parameters like superficial tension, adhesive and cohesive interactions, or spreading implications in the behavior of multicomponent systems as microemulsions. The equilibrium established between adhesive and cohesive forces governs the displaying and wettability of microemulsions at a given surface, being strongly associated with a variation in the formulation factors. The goniometric technique offers a reliable solution in assessing surface characteristics for nanocolloids, by dealing with the *pendant drop* and *contact angle models,* which were mathematically described using Young-Laplace and Young equations, as previously reported [63,103,104].

In the case of miconazole-based microemulsions, superficial tension (ST) was tested using both analysis models, and the results obtained are presented in Table 8. According to the pendant drop model, the shape of a drop dispensed through a Hamilton syringe needle tip was analyzed considering the pressure difference at the liquid/air interface, the radii of curvature, and the surface tension opposing the gravitational force. Thus, the mean values of ST varied between 17.97 ± 0.62–25.80 ± 0.03 mN/m. A slight reduction in γLG was observed in the group of viscous ME 5–ME 8 microemulsions, and the minimum value was obtained for ME 10.

In contrast, by applying the sessile drop method, the mean values for γ_LG_ depended on composition. In this case, γ_LG_ is specific for drops displayed on a solid surface, with ST values placed between 5.65 ± 0.014–44.53 ± 1.058 mN/m, which assumed different behaviors in matters of adhesion and spreading. The highest values between 26.48–44.53 mN/m were obtained for microemulsions described by high contact angles of 48.00–57.75° (ME 1–ME 4, and ME 9) where the superficial tension opposed to an increased wetting of the surface. A high work of adhesion was calculated in this case and varied between 42.23–74.77 mN/m. The work of cohesion attained maximum values of 52.95–89.05 mN/m, signifying strong interactions between microemulsion molecules. The phenomenon was also defined by high negative values of the spreading coefficient varying between −10.72 and – 44.52 mN/m.

Secondly, it was well observed that low values of γ_LG_ were obtained in the case of gel-like ME 5–ME 8 and ME 10 microemulsions, with a significant variation between 5.65 ± 0.014–10.11 ± 0.095 mN/m. A substantial decrease in ST was strongly correlated to the high oil content selected in the preparation process, which may highly impact the wettability and spreading of the formulation at the surface of administration. In this group, the contact angle values varied between 37.03–51.10°, with a wettability behavior influenced by the oil phase and also through the fluidizer effect of PEG 400 that increased the hydrophilicity of the samples. Accordingly, the work of adhesion quantified in values of 9.84–16.44 mN/m sustained a low adhesive effect but also a decrease in cohesion forces from 20.22 mN/m to 11.29 mN/m, and an increase in the spreadability with low negative values, closer to zero, from −3.78 mN/m to −1.19 mN/m, as it can be viewed in Table 9.

In the study of Wang W. et al., microemulsions formulated with fatty acid methyl esters were explored with attention to superficial properties. The dynamic contact angle was useful to appreciate the spreading phenomenon of diluted microemulsions at the surface level. Moreover, it was stated that the superficial tension of the dispersion decreased when the microemulsions were tested as concentrated systems [104], and this may explain the behavior of the gel-like microemulsions. A decrease in surface tension is explained by the activity of surfactant and cosurfactant in promoting negative free energy at the oil/water interface [105,106].

**Figure 8:**
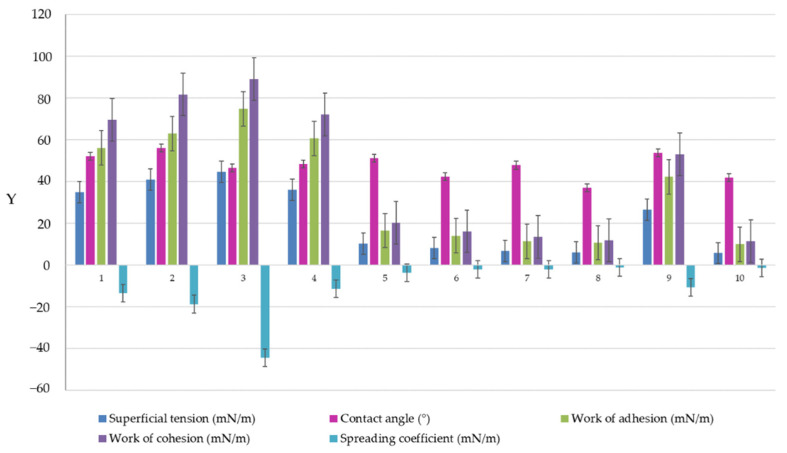
Variation in superficial parameters for the ME 1–10 microemulsions, evaluated following the sessile drop method at 24 ± 0.5 °C.

It can be appreciated that the microemulsion samples experienced partial wetting, having θ values under 90°, and the most hydrophilic microemulsion was ME 8 formulated with oleic acid 10%, Tween 20 40%, and PEG 400 20%. The variation for each parameter analyzed through the sessile drop method can be observed in Figure 8 comparatively. The ordinate axis is noted with “y” labeling by turning the experimental values of superficial tension, contact angle, work of adhesion, work of cohesion, and work of spreading. The best spreading was observed for the gel-like microemulsions formulated with the maximum concentration of oleic acid.

### 3.11. In Vitro Drug Release

**Figure 9:**
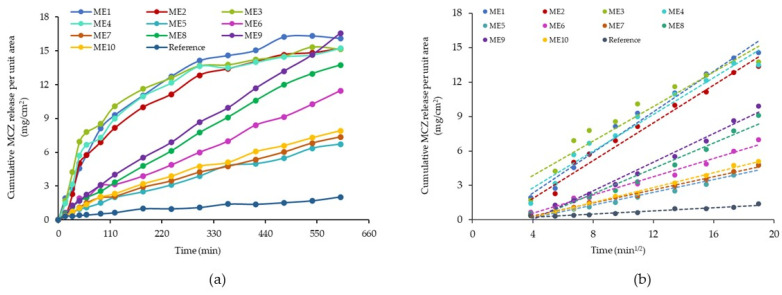
MCZ release profiles—(**a**) Cumulative amount released (mg) per unit area (cm^2^) as a function of time (min) for the microemulsions ME 1-ME 10, and (**b**) Identification of Higuchi model’s linearity range for cumulative MCZ release per unit area (mg/cm^2^) as a function of the square root of time.

The biopharmaceutical analysis of the miconazole-based microemulsions valued the presence of significant formulation factors in the drug diffusion process. The presence of the oleic acid and PEG 400 had an essential contribution in explaining diffusion phenomena (*p* < 0.05), as the statistical analysis confirmed over 3.11.3. section. The miconazole release profiles defined as the cumulative amount released (mg) per unit area (cm^2^) as a function of time (min) are presented in Figure 9—case (a). The release of miconazole during 600 min was evaluated for different behaviors that collided with the structure of the fluid and gel-like microemulsions. In the case of the fluid ME 1–ME 4 containing oleic acid 5%, a burst release was seen in the first 2 h, followed by a gradual liberation up to 10 h. In contrast, ME 9, an intermediary point in the present design, had a release profile resembling the gel-like systems. In this case, its behavior can be justified by an increased oil phase content of 6.25% with a strong effect upon drug release and a modified profile allure. As can be seen in the cases of ME 5–ME 8 and ME 10, the obtained profiles suggested a controlled drug release. ME 5 and ME 7 presenting PEG 400 10% were characterized by an inferior release. Meanwhile, ME 6 and ME 8 containing PEG 400 20% exceeded a drug release quantified by a cumulative release per unit area up to 12 mg/cm^2^. MCZ release from microemulsions was superior to the commercial gel, which assumed a constant profile of MCZ diffusion.

Kinetic descriptors specific for MCZ release from the ME 1–ME 10 microemulsions and the reference gel were described as diffusion coefficients and correlation coefficients (R) that well fitted the Higuchi model. The results are presented in Table 9, in relationship with the individual profiles of in vitro miconazole release from microemulsions after applying the Higuchi model. Generally, the kinetic model was used to describe and validate the kinetic release of various drugs from designed and studied microemulsions with ibuprofen [89], quercetin [52], oleanolic acid [107], or from more complex systems containing microemulsions for delivery of clotrimazole from nanofibers [108], polymer-based gels, microemulsion-based gels, or liposomal gels with croconazole [109]. In this study, the cumulative amount of MCZ released per unit area (mg/cm^2^) was graphically presented as a function of the square root of time, and the linear profiles validating the model were projected (Figure 9—case b).

The diffusion coefficients varied between 0.0162·10^−5^ cm^2^/s (in the case of the reference gel) and 2.56·10^−5^ cm^2^/s in the case of ME 1, which exhibited the highest release along with ME 2 and ME 4. From the group of the gel-like formulations, ME 8 had the maximum diffusion coefficient value of 1.00 cm^2^/s.

The structural complexity of the microemulsions governs the drug release, being composition-dependent. Thereby, the release was superior from all the microemulsions compared to the gel system. The observation is closely related to some reports where the reference was a miconazole suspension tested using dialysis membranes [71,72] or a cream-based formulation with miconazole analyzed through permeation studies [110]. Several factors affecting drug diffusion are related to the solubility of miconazole in the aqueous and the lipophilic phase, the stabilizer effect of Tween 20 and PEG 400, and particular dynamics occurring in the formation of fluid and viscous microemulsion [47]. Thereby, droplet size and viscosity are the main physical parameters with a direct impact on the drug release. A high level of the aqueous phase and the nanometer scale of the dispersed droplets sustain a faster diffusion, as previously described for microemulsions with clotrimazole [111]. Reduced droplet diameters between 119.60–188.33 nm, specific for the ME 1–ME 4 fluid systems, were correlated to higher values of diffusion coefficients, while in the case of ME 5–ME 8 gel-like systems, elevated sizes up to 250.20 nm slowed down the MCZ release. It was observed that an increase in drug diffusion is linked to a partition of the API in the two solubilizers—the oil phase and the surfactant/cosurfactant mixture, determining the API to reside at the oil/water interface, accelerating diffusion [98]. As the oil concentration increases, the API becomes encapsulated in oily cores, implied in strong interactions that reinforce the internal structure and increase the viscosity, promoting a controlled drug release [49].

### 3.12. Statistical Analysis for the Miconazole Microemulsions Using 2^3^ Full Factorial Design

The last step of the study comprised the application of a Quality by Design (QbD) approach to assess microemulsions with adequate critical quality attributes. Beginning with the projection of the 2^3^ + 2 factorial model with 10 experimental runs generated in Design Expert, the three formulation factors noted X_1_: Oleic acid (%), X_2_: Tween 20 (%), and X_3_: PEG 400 (%) were analyzed as independent variables that may influence the variation of three main responses namely Y_1_: mean droplet size—Ds (nm), Y_2_: work of adhesion—W_a_ (mN/m), and Y_3_: diffusion coefficient—D (cm^2^/min).

Response surface regression was based on a statistical interpretation of responses using multiple linear regression combined with response surface methodology [112,113]. Using ANOVA analysis, it was interpreted the statistical significance of independent factors and possible interactions that may occur by analyzing the *p*-value for each model, together with the lack of fit value, R^2^, and adjusted R^2^, by assuming *p* < 0.05 as desired for the significance of the statistical model, and *p* > 0.05 for the lack-of-fit. Moreover, a critical interpretation using contour plots, surface response plots, and interaction plots was proposed to visualize formulation factors’ influence on responses [63,112,114].

The experimental design is of outstanding relevance to assess the complexity of the microemulsions and find out a desired system that must accomplish a sum of criteria relying on their pharmaceutical applicability for buccal application: a reduced droplet size, an adequate work of adhesion, and a high diffusion coefficient that assumes a high release rate of miconazole to support a sustained action on oral lesions. In this sense, a predictive optimization was implemented to find solutions with the best desirability coefficients.

Table 10 presents the independent and dependent variables analyzed using the design of the experiment for the ten systems generated through the full factorial plan. The experimental values of the three responses are accompanied by predicted values proposed over the statistical analysis.

#### 3.12.1. Statistical Interpretation for Mean Droplet Size

**Figure 10:**
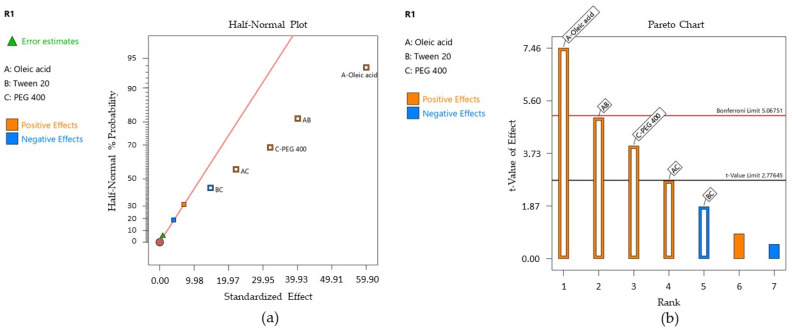
Initiative step in mathematical modeling of Y_1_ response using the Half-Normal Plot of the standardized effect—case (**a**), and Pareto Chart ranking the significant terms as a function of the t-value and Bonferroni limit—case (**b**).

Droplet size has a real impact on the pharmaceutical properties of the microemulsions, influencing other intrinsic attributes like viscosity, adhesion, and spreadability, as well as the release of the active ingredient at the area of treatment [63]. By appreciating the nanometric size of the microemulsion droplets, it can be estimated a high surface area influenced by dynamics in the molecular arrangements of surfactant and cosurfactant at the oil/water interface that promotes a highly reduced interfacial tension and an increased contact at the level of the buccal mucosa [115]. In this sense, a statistical interpretation for the mean Ds of the projected microemulsions was proposed to find how formulation variables influence the hydrodynamic diameter. The mathematical interpretation of the Y_1_ response was initially created using the Half-Normal plot of the standardized effect and the Pareto chart that well-defined the significance of each term for the statistical model [52], being presented in Figure 10—cases (a) and (b).

According to the response surface regression analysis, Ds was significantly influenced by oleic acid (%)—X_1_ factor, PEG 400 (%)—X_3_ factor, and the X_1_X_2_ interaction, with *p* < 0.05. The projected model was significant, with a *p*-value of 0.0150 and an F value of 21.29. There is only a 1.50% chance that an F value this large could occur due to noise. The polynomial equation that fitted the Y_1_ response is presented herein (Equation (5)):

Y_1_ = 163.92 + 29.95 X_1_ + 16.02 X_3_ + 20.45 X_1_X_2_ + 11.30 X_1_X_3_ − 7.58 X_2_X_3_ (8)

The experimental assessments through DLS revealed that the mean Ds values varied between 119.60 nm and 250.20 nm, being influenced by the oil phase, PEG 400, and the interaction between oleic acid and Tween 20. It can be observed that the X_1_ term had a strong positive effect in terms of increasing Ds. The same observation was valid for the X_3_ term described by a positive coefficient in the same manner the X_1_X_2_ interaction has shown. The R^2^ obtained for the model was 0.9726, while the adjusted R^2^ by reference to degrees of freedom was 0.9269. Table 11 exposes the terms analyzed in the ANOVA test as a function of F and *p*-values at a confidence level of 95%.

Similar assessments were obtained in a full factorial design to develop nanostructured lipid carriers with salicylic acid. The multiple linear regression was defined by significant factors (surfactant concentration and the ratio of the lipidic phase) implied in particle size variation in an opposite manner [116].

**Figure 11:**
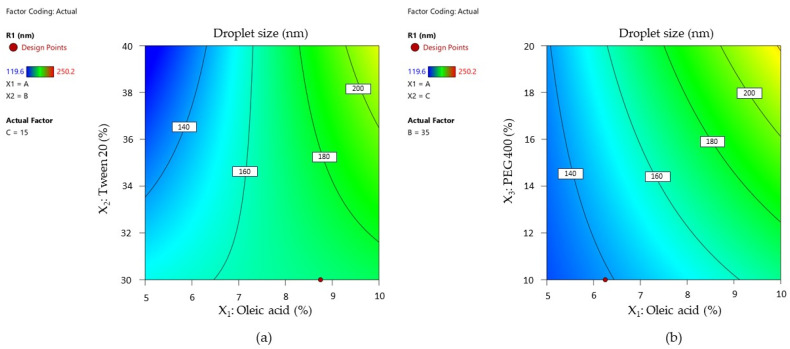
Contour plots for mean droplet size response (Y_1_) as a function of (**a**) X_1_: Oleic acid (%), X_2_: Tween 20 (%), and (**b**) X_1_: Oleic acid (%), X_3_: PEG 400 (%).

Response variation as a function of independent variables was graphically described by designing contour plots of response. The representations are shown in Figure 11—cases (a) and (b).

The two contour plots represented below showed how droplet size diameter changed with the increase in oleic acid from 5% to 10% as a function of the Tween 20 variation (Figure 11—case (a)). The interaction of Tween 20 with oleic acid can be observed only in low concentrations of the oil phase, where droplet size varies between 120–160 nm. Over 160 nm, oleic acid has a dominant effect on particle size, and the contour lines tend to become parallel with the axis of the X_2_ term.

The droplet size is differently represented in Figure 11—case (b), where at low concentrations of oleic acid and PEG 400, Ds is concentrated under 140 nm, and it tends to increase with the increase in their concentration. Even PEG 400 promotes a fluidizing effect with increasing concentration, and it is implied in droplets growing at the maximum oil concentration.

**Figure 12:**
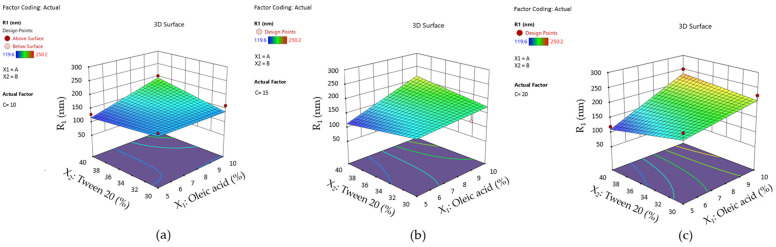
Surface plots for mean droplet size response (Y_1_) as a function of X_1_: Oleic acid, X_2_: Tween 20, by fixing PEG 400 concentration from 10%—case (**a**), to 15%—case (**b**), up to 20%—case (**c**).

These effects can be better visualized using surface plots for the mean droplet size response represented as a function of the X_1_ and X_2_ terms by variating PEG 400 concentration, as shown in Figure 12. From case (a) to (c), it can be observed that variation in droplet size tends to exceed a high limit with the increase in PEG 400 (%).

**Figure 13:**
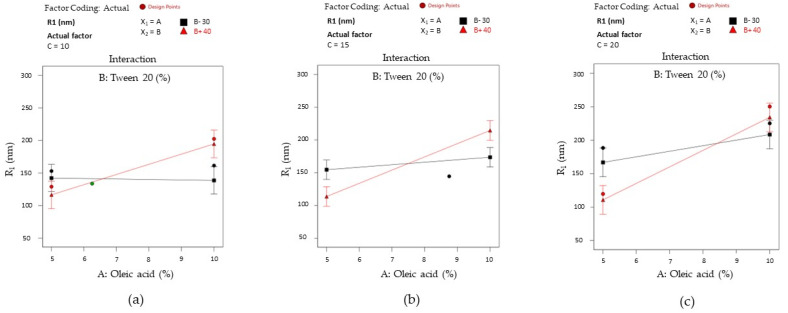
Interaction plots for X_1_X_2_ term, considering three cases: (**a**) X_1_X_2_ interaction when X_3_ is fixed at 10%; (**b**) X_1_X_2_ interaction when X_3_ is fixed at 15%; (**c**) X_1_X_2_ interaction when X_3_ is fixed at 20%.

The results were accompanied by interaction plots for the X_1_X_2_ term of the equation and are presented in Figure 13. The interactions were graphically described using the same principle. According to case (a), when PEG 400 (noted as C) is fixed at 10%, two situations can be visualized. With the variation of oleic acid from 5% to 10%, the X_2_ factor (at 30%) did not affect droplet size variation, being constantly settled around 150 nm. If Tween 20 is fixed at 40%, the response takes ascending values from an inferior level up to approximately 200 nm. The slope represented in this case with a red line crosses the black line, defining an intersection where the output remains the same even if Tween 20 has minimum or maximum levels.

The reversal style of the interaction is also maintained in case (b), where the actual factor X_3_ (noted with C) was fixed at 15%. Consequently, it can be seen that at the minimum concentration of the X_1_ and X_2_ terms, the response is placed around 150 nm, being balanced up to 175 nm if the X_1_ term is considered a maximum point. At the maximum level of Tween 20, kept with a minimum level of the oil phase, the output diminishes through 100 nm, but it is drastically modified at the maximum oil level.

In the last case (c), when PEG 400 (noted as C) was fixed at 20%, it can be observed a large output domain from 100 nm to approximately 200 nm with the variation of X_2_ term from the low to the high level, keeping X_1_ as minimum. A narrowing of the output domain was seen in the opposite case, at the increase in the oil level. When the oil phase and PEG 400 attain the maximum level, any of the two concentrations of Tween 20 will reduce the Ds response.

#### 3.12.2. Statistical Interpretation for Work of Adhesion

Work of adhesion represents a superficial parameter that connects physico-chemical characteristics like superficial tension and contact angle with their in-depth implication in drug delivery. The work of adhesion represents the energy implied in the separation process of a liquid material from a solid substrate [117]. In this case, an important aspect is referred to the displaying of the formulation in the area of administration, which can be obtained with a high adhesion, a lower cohesive force, and spreading coefficients that are desired to tend through zero or positive values to attain a complete wetting of the formulation [118,119].

**Figure 14:**
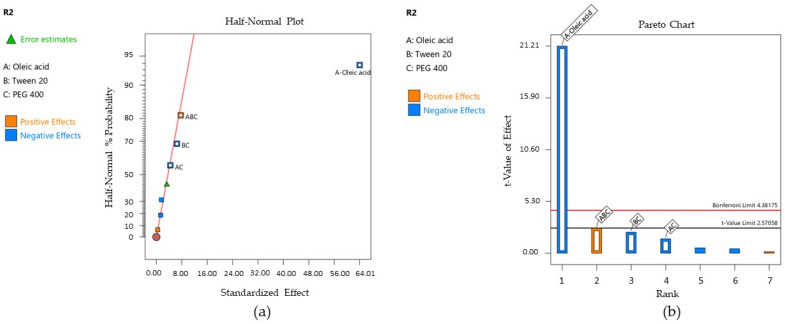
Initiative step in mathematical modeling of Y_2_ response using the Half-Normal Plot of the standardized effect—case (**a**), and Pareto Chart ranking the significant terms as a function of the t-value and Bonferroni limit—case (**b**).

Over the response regression analysis, W_a_ was significantly influenced only by the oleic acid—X_1_ factor, with *p* < 0.05. The Half-Normal Plot of the standardized effect and Pareto chart representing the terms of priority to generate the model are further exposed in Figure 14—cases (a) and (b), where X_1_ was coded with A, X_2—_with B, and X_3—_with C for both the isolated terms and their interactions.

The projected model was significant, with a *p*-value of 0.0002 and an F value of 115.37. The reduced polynomial equation that fitted the response is presented below, and the results of the ANOVA analysis are listed in Table 12. The model was validated by a regression coefficient R^2^ of 0.9914 and an adjusted R^2^ of 0.9825.

Y_2_ = 39.71 − 32.00 X_1_ − 2.26 X_1_X_3_ − 3.34 X_2_X_3_ + 3.96 X_1_X_2_X_3_ (9)

The mathematical modeling of the work of adhesion was performed using the Young–Dupré equation. Thus, considering the results placed between 11.29–89.05 mN/m, it was appreciated that the oleic acid term (X_1_) negatively affected the adhesion properties of the microemulsions as a function of its concentration. The lowest values for the work of adhesion were obtained when oleic acid was selected in a concentration over 5%.

**Figure 15:**
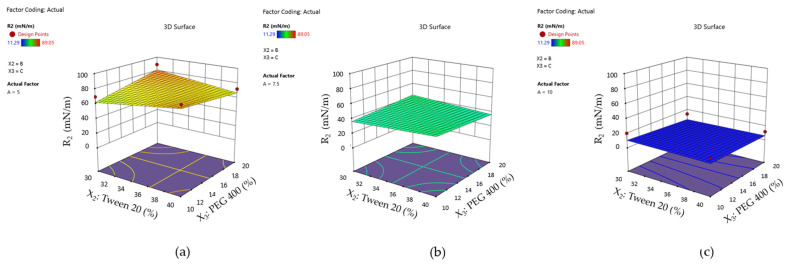
Surface plots for work of adhesion (Y_2_) as a function of X_2_: Tween 20 (%), X_3_: PEG 400 (%), by fixing oleic acid concentration from 5%—case (**a**), to 7.5%—case (**b**), up to 10%—case (**c**).

**Figure 16:**
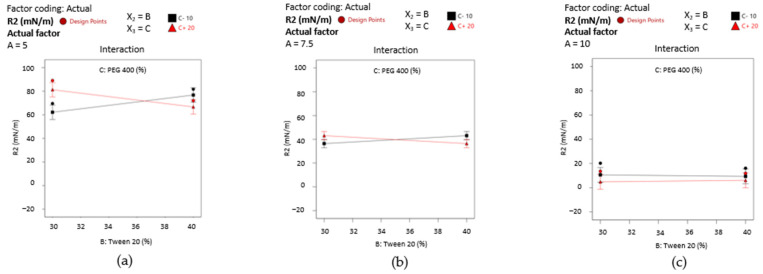
Interaction plots emphasizing the main effect of X_1_ term: (**a**) X_2_X_3_ interaction when X_1_ is fixed at 5%; (**b**) X_2_X_3_ interaction when X_1_ is fixed at 7.5%; (**c**) lack of X_2_X_3_ interaction when X_1_ is fixed at 10%.

By projecting the surface responses for Y_2_ as presented in Figure 15—cases (a)-(c), it can be emphasized the variation in the work of adhesion from high values—case (a) through the lowest ones—case (c), by assuming that X_2_ and X_3_ factors did not influence its variation. The surface is moved along the scale in the same manner as the interaction plots presented in Figure 16—cases (a)–(c) show the placement of the response values in three main groups.

Firstly, it can be mentioned the domain between 60–80 mN/m from Figure 16—case (a), where the oleic acid term (noted with A) is fixed at 5%. There is only one case where a slight variation in the output can be observed, but just in the specified range when the X_2_ term and X_3_ term are modified. Thus, Tween 20 (40%) determined an increase in the work of adhesion when PEG 400 is considered 10%, or in reverse, a decrease in response when PEG 400 is considered 20%. When the oil content is increased to 7.5%—case (b), the output range drops down to around 40 mN/m, where the significance of the X_2_ and X_3_ factors is lowered. The most substantial effect is seen in case (c), where the lines tend to be straight and uncrossed alongside the X_2_ or X_3_ axis, and an output concentrated under 20 mN/m).

#### 3.12.3. Statistical Interpretation for Diffusion Coefficient of Miconazole

The response regression analysis for the diffusion coefficient (D) followed a particular pathway compared to the previously performed designs. Thus, a square root transformation was applied to obtain a linear regression for a relevant model that can better fit the response. Over the first initial configuration of the model with linear regression with no transformation, the Box–Cox plot helped in diagnosing inadequacies of the statistical model by proposing a square root transformation. Koliqi R. et al. used this algorithm to obtain an improved predictive model for studying the entrapment efficiency and drug content in the development of polymeric nanoparticles [120]. The transformation approach is helpful in the experimental design when it is wisely applied to control the response variance [121,122].

**Figure 17:**
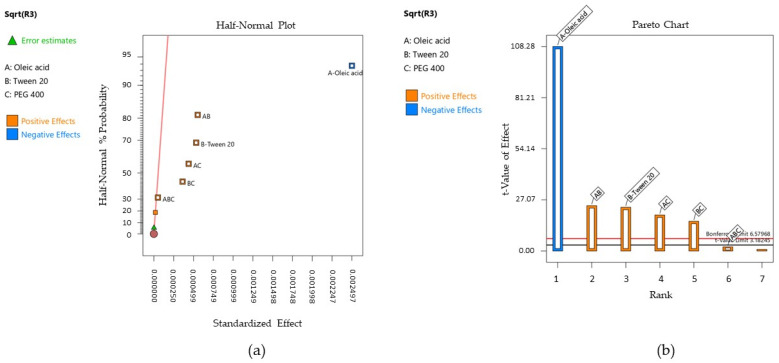
Initiative step in mathematical modeling of Y_3_ response using the Half-Normal Plot of the standardized effect—case (**a**), and Pareto Chart ranking the significant terms as a function of the t-value and Bonferroni limit—case (**b**).

The Half-Normal Plot of the standardized effect and the Pareto chart described the significance of the terms and are presented in Figure 17—cases (a) and (b).

Following the response surface regression analysis, the diffusion coefficient was significantly influenced by oleic acid (%)—X_1_ factor, Tween 20 (%)—X_2_ factor, and their interactions between the factors X_1_X_2_, X_1_X_3_, and X_2_X_3_, with a *p* < 0.05. The new model was significant, with a *p*-value of 0.0004 and an F of 2362.10. There is a 0.04% chance that an F value this large could occur due to noise. The regression equation (Equation (7)) obtained through the square root transformation fitting the Y_3_ response is further presented, together with the results of the ANOVA analysis listed in Table 13. The model was confirmed by regression coefficients R^2^ of 0.9999 and an adjusted R^2^ of 0.9994.

Sqrt(Y_3_) = 0.0031 − 0.0012 X_1_ + 0.0003 X_2_ +0.0003 X_1_X_2_ + 0.0002 X_1_X_3_ + 0.0002 X_2_X_3_ (10)

Considering the experimental results, drug diffusion was characterized by diffusion coefficients between 0.252·10^−5^ cm^2^/s and 2.56·10^−5^ cm^2^/s, values that are closely related to the main effects described in the regression analysis. It was observed in this case that the oleic acid (%)—X_1_ term had a negative effect on drug diffusion, while the X_2_ term and the three double interactions were significant for a positive effect on drug release.

**Figure 18:**
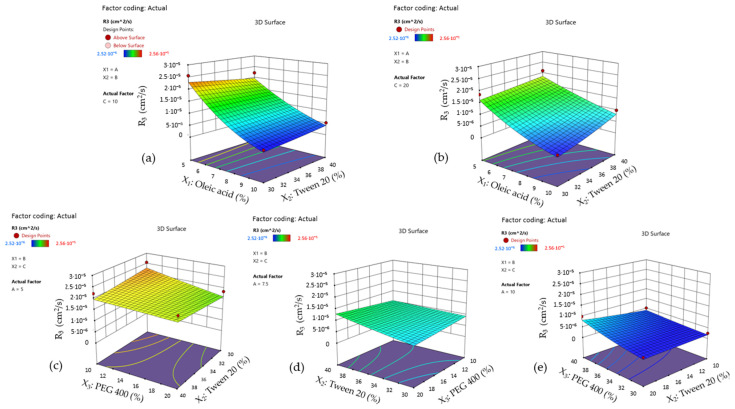
Surface plots for diffusion coefficient (Y_3_) as a function of X_1_: Oleic acid (%), X_2_: Tween 20 (%) terms, by fixing PEG 400 concentration at 10%—case (**a**), 20%—case (**b**); and as a function of X_2_: Tween 20 (%), X_3_: PEG 400 (%), by fixing oleic acid concentration at 5%—case (**c**), 7.5%—case (**d**), and 10%—case (**e**).

A graphical interpretation for the statistical analysis and significant factors affecting drug diffusion (Y_3_) response was implemented using surface plots, as can be seen in Figure 18—cases (a)–(e). In the first case (a), where the X_3_ term was fixed at a minimum level, the MCZ diffusion coefficient reached a maximum point as long as the oil phase (%)—X_1_ term was fixed at 5%. A decline can be well observed when the X_1_ term tended to vary through 10%, without any interference from the X_2_ term. The contour lines were oriented in a perpendicular manner on the X_1_ axis.

The situation was changed in the case (b). After setting the X_3_ term at 20%, the profile of the response surface was changed, and a slight decrease in drug diffusion and a dependence of the response upon the two factors can be noticed. Thus, in this case, the contour lines are oriented through both the X_1_ and X_2_ axis. At the minimum concentration of the oil phase and the maximum level of Tween 20, diffusion coefficients are magnified, determining a rapid release. Intermediary results were obtained when variating the oil content through middle values and keeping surfactant content at the maximum level. In contrast, an opposite effect is achieved when a higher oil phase is stabilized with a lower concentration of the stabilizer.

Furthermore, in Figure 18—cases (c)–(e), the variation of the diffusion coefficient was observed from a different perspective, taking into consideration the primary axis for X_2_ and X_3_ terms dynamics under the effect of progressive modification of the oil phase (%) from 5% to 10%. The profiles preserved a similar allure as previously presented over the statistical analysis for the Y_2_ response.

**Figure 19:**
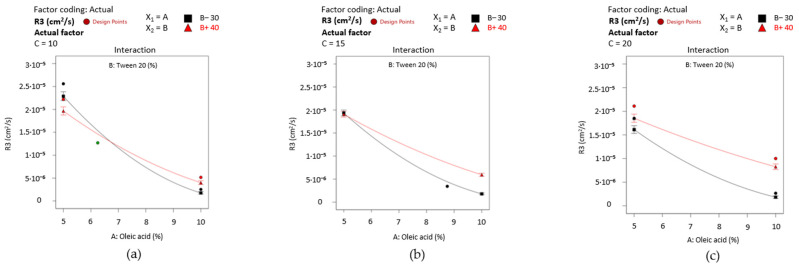
Interaction plots emphasizing X_1_X_2_ term when X_3_ term is fixed at 10%—case (**a**), at 15%—case (**b**), up to 20% specific for lack of interaction—case (**c**).

For final appreciations, interaction plots were represented in Figure 19—cases (a)–(c) to emphasize the particular effects of the formulation factors with an accent on the X_1_X_2_ effect on drug diffusion when X_3_ was selected on the 10–20% interval. At any variation of PEG 400, drug diffusion was delayed when oleic acid (%) increased to 10%. A favorable dynamic was seen when PEG 400 was fixed at 20%. Tween 20 40% may interfere in drug diffusion, obtaining intermediary values of D (cm^2^/s), even at higher levels of the oil phase.

It can be stated that the fluid-like ME 1–ME 4 microemulsions prepared with oleic acid 5% exhibited a higher drug release with maximum diffusion coefficients (1.85·10^−5^–2.56·10^−5^ cm^2^/s) and adequate adhesion (56.07–89.05 mN/m). Taking into consideration the presence of some instability phenomena in the case of ME 1, ME 3, and ME 9 (as pointed out in the organoleptic analysis), stability studies will be proposed in future studies to evidentiate the thermodynamic stability of microemulsions. In the present case, it was valued the contribution of ME 2 and ME 4 as model systems that can solubilize and enhance miconazole release. Similarly, the two microemulsions are characterized by nano-sized droplets under 200 nm, directly influencing the kinetic mechanism and the adhesion properties. To promote a controlled release and a higher MCZ liberation, the gel-like ME 8 microemulsion can satisfy these conditions due to its maximum concentration of PEG 400 20% and Tween 20 40%. Even if the two stabilizers impacted the droplet size by increasing it up to 250 nm, creating gel-like structures became a promising approach for further research of mucoadhesive systems with tailored physico-chemical and biopharmaceutical attributes. ME 10 has distinguished itself from the group as a gel-like microemulsion with a superior consistency index and an average droplet size of 144.36 nm but with a reduced drug diffusion (3.43·10^−6^ cm^2^/s) and lower adhesion (11.29 mN/m).

Implementation of new factorial designs inspired by the need to develop new platforms for drug delivery by using old molecules with improved therapeutic profiles, like antifungals. A 2^3^ full factorial design was proposed to optimize PLGA nanoparticles with itraconazole by analyzing the impact of PLGA, benzyl benzoate, and the drug as formulation factors influencing particle size [123]. The combination of formulation design with mathematical modeling creates an excellent opportunity to rigorously characterize and describe the intimate contribution of the formulation factors for pharmaceutical-relevant attributes like droplet size, work of adhesion, and drug diffusion.

#### 3.12.4. Optimization of Miconazole-Based Microemulsions

Droplet size was a critical parameter influenced by the oil phase (%), PEG 400 (%), and the interaction between the oil and Tween 20. Minimizing the oil level represents a primary condition for optimizing the formation of microemulsions with reduced droplet size, rapid diffusion, and a higher work of adhesion. ME 4 properties were found to agree with these requirements, being considered a model system.

On the other side, an increase in the oil phase carries itself a better wettability, a good spreading determined by smaller contact angles, a high stability promoted by the creation of strong networks specific for gel-like systems, but in reverse, a poor adhesion. The higher droplet growth delayed the drug diffusion. Response transformation using the square root algorithm offered consistent information revealing the contribution of the oleic acid for drug release and the particular interaction between the three excipients.

**Figure 20:**
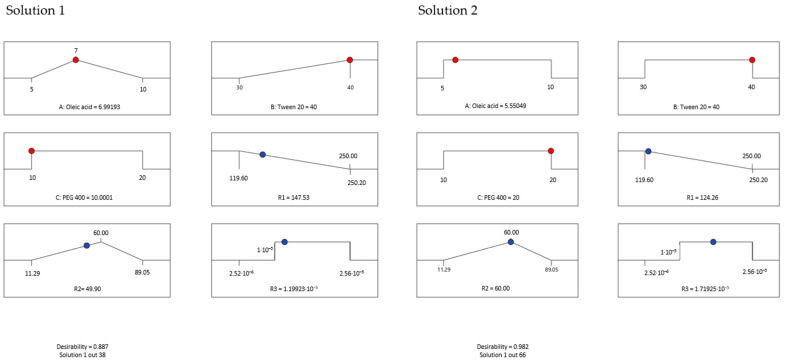
Predictive optimization to depict two optimized solutions for microemulsions with adequate critical attributes.

Starting from the leading-edge systems (ME 4, ME 8, and ME 10) characterized over the study, a numerical optimization was implemented to find microemulsions with reduced droplet size, moderate adhesion, and high diffusion coefficient. In this final stage, the parameters were fixed using constraints to achieve solutions with high desirability coefficients [124], as seen in Table 14. The solutions obtained using the predictive tool of the software are presented in Figure 20.

In the first case, for a microemulsion containing oleic acid 6.99%, Tween 20 40%, and PEG 400 10%, the predicted responses proposed a mean droplet size of 147.53 nm, a work of adhesion of 49.90 mN/m, and a drug diffusion coefficient of 1.19·10^−5^ cm^2^/s, with a good desirability of 0.8870.

The second solution obtained over the analysis described a potential microemulsion in which miconazole can be solubilized using oleic acid 5.55%, with droplets stabilized using Tween 20 40% and PEG 400 20%. The system is characterized by predictive responses that assume a mean droplet size of 124.26 nm, a work of adhesion of 60 mN/m, and a maximum diffusion coefficient of 1.719·10^−5^ cm^2^/s, with the best desirability coefficient of 0.9820.

The predictive optimization results offered a promising perspective on developing nanostructured platforms for drug delivery that can be further designed to promote antifungal activity in oral candidiasis.

## 4. Conclusions

The present study revealed an open pathway for the development process of new microemulsions for local therapy in oral candidiasis. In this context, miconazole base was the antifungal model drug studied to improve its solubility and bioavailability using nano-sized microemulsions composed of oleic acid 5–10%, Tween 20 30–40%, and PEG 400 10–20%. The formulation study was combined with a 2^3^ full factorial design with two lack of fit points. Designed as fluid and gel-like systems, the microemulsions were characterized from physical and biopharmaceutical perspectives and further researched for pharmaceutical applications in buccal delivery.

The characterization process was accompanied by statistical analysis, succeeded by response surface methodology to optimize microemulsions with adequate critical quality attributes.

The connections found between physical and biopharmaceutical attributes conceived a promising algorithm to optimize microemulsions that can exhibit a reduced droplet size, a moderate adhesion up to 60 mN/m, and superior diffusion, being inspired by three systems resulting from the factorial plan.

A microemulsion containing oleic acid 6.99%, Tween 20 40%, and PEG 400 10%, with predicted responses quantified as mean droplet size of 147.53 nm, a work of adhesion of 49.90 mN/m, and a drug diffusion coefficient of 1.19·10^−5^ cm^2^/s was a first solution of the predictive optimization for a model microemulsion.

The second solution obtained over the analysis described a potential fluid microemulsion in which miconazole can be solubilized using oleic acid 5.55%, with droplets stabilized using Tween 20 40% and PEG 400 20%. The system was characterized by predictive responses that assume a mean droplet size of 124.26 nm, a work of adhesion of 60 mN/m, and a maximum diffusion coefficient of 1.719·10^−5^ cm^2^/s. The last solution resembled the model microemulsion described in the research, but in this case, a slight increase in the oil content can modulate the response parameters to the desired constraints, resulting in promising performant systems that may impose their quality attributes in buccal drug delivery.

## Figures and Tables

**Table 2:** Composition of the microemulsions obtained using an augmented 2^3^ full factorial plan.

Formulation	Oleic Acid (%) X_1_	Tween 20 (%) X_2_	^1^ PEG 400 (%) X_3_	Water (%)	^2^ MCZ (%)
ME 1	5	30	10	53	2
ME 2	5	40	10	43	2
ME 3	5	30	20	43	2
ME 4	5	40	20	33	2
ME 5	10	30	10	48	2
ME 6	10	40	10	38	2
ME 7	10	30	20	38	2
ME 8	10	40	20	28	2
ME 9	6.25	35	10	51.75	2
ME 10	8.75	30	15	44.25	2

^1^ PEG 400 represents polyethylene glycol 400, and ^2^ MCZ—miconazole.

**Table 3:** Solubility values for eight excipients obtained through the spectrophotometric technique.

Components	Excipient	Solubility (mg/mL)
Oils	Isopropyl myristate	36 ± 3
	Oleic acid	110 ± 5
Surfactants	Tween 80	152 ± 10
	Tween 20	236 ± 11
	Kolliphor 407	27 ± 5
Cosurfactants	Propylene glycol	193 ± 7
	Polyethylene glycol 200	179 ± 12
	Polyethylene glycol 400	209 ± 13

**Table 4:** Factorial plan with 2 levels and 3 factors as independent variables.

Factor	Variable	Level	
		Low (−1)	High (+1)
X_1_	Oleic acid (%)	5	10
X_2_	Tween 20 (%)	30	40
X_3_	PEG 400 (%)	10	20

**Table 5:** Experimental matrix equivalent to a 2^3^ + 2 full factorial model for microemulsion design.

Std.	Block	Run	X_1_	X_2_	X_3_
			Oleic Acid (%)	Tween 20 (%)	PEG 400 (%)
1	1	1	−1	−1	−1
3	1	2	−1	+1	−1
5	1	3	−1	−1	+1
7	1	4	−1	+1	−1
2	1	5	+1	−1	−1
4	1	6	+1	+1	−1
6	1	7	+1	−1	+1
8	1	8	+1	+1	+1
9	2	9	0.625	0	−1
10	2	10	0.875	−1	0

**Table 6:** Physico-chemical parameters of the O/W microemulsions determined at 24 ± 0.5 °C (*n* = 3).

Code	pH	Conductivity (μS/cm)	Refractive Index	Z_average_ (nm)	PDI	D_10%_ (nm)	D_50%_ (nm)	D_90%_ (nm)	Span	Zeta (mV)
ME1	5.29 ± 0.01	101.90 ± 0.66	1.3823 ± 0.0002	152.89 ± 2.10	0.230 ± 0.001	118.11	205.71	375.23	1.24	+10.76 ± 1.15
ME2	5.60 ± 0.01	88.70 ± 0.10	1.4047 ± 0.0001	128.90 ± 2.15	0.303 ± 0.002	98.17	187.54	375.23	1.47	+10.68 ± 1.19
ME3	5.53 ± 0.07	55.70 ± 0.30	1.4012 ± 0.0001	188.33 ± 5.03	0.296 ± 0.011	142.11	284.33	543.17	1.41	+14.00 ± 1.28
ME4	5.74 ± 0.02	47.60 ± 0.10	1.4238 ± 0.0001	119.60 ± 1.37	0.332 ± 0.002	93.73	187.54	358.28	1.41	+10.68 ± 2.19
ME5	5.15 ± 0.02	21.67 ± 0.06	1.3959 ± 0.0001	161.34 ± 4.06	0.165 ± 0.003	123.7	205.71	342.09	1.06	+12.34 ± 1.42
ME6	5.48 ± 0.01	18.07 ± 0.06	1.4154 ± 0.0001	202.29 ± 5.02	0.292 ± 0.002	155.87	297.78	568.88	1.38	+9.62 ± 3.23
ME7	5.27 ± 0.01	7.96 ± 0.01	1.4148 ± 0.0001	225.13 ± 5.20	0.280 ± 0.005	170.9	326.63	623.99	1.38	+13.30 ± 1.07
ME8	5.80 ± 0.01	8.80 ± 0.25	1.4318 ± 0.0001	250.20 ± 6.50	0.301 ± 0.007	196.41	375.23	716.84	1.38	+12.62 ± 1.12
ME9	5.57 ± 0.01	74.43 ± 0.03	1.3895 ± 0.0001	133.57 ± 3.08	0.214 ± 0.012	102.81	179.07	311.87	1.16	+9.10 ± 1.94
ME10	5.48 ± 0.00	18.89 ± 0.02	1.4058 ± 0.0001	144.36 ± 1.71	0.230 ± 0.005	112.77	196.41	358.28	1.24	+12.85 ± 2.44

**Table 7:** Rheological descriptors describing flow behavior for the microemulsions tested at 37 ± 0.5 °C.

Code	Viscosity (Pa·s)/Consistency Index (Pa·s^n^)	^1^ n	^2^ R	Rheological Model
ME 1	1.001	0.49	0.9974	Ostwald–de Waele
ME 2	0.346	1.0	0.9993	Newton
ME 3	0.192	1.0	0.9998	Newton
ME 4	0.304	1.0	0.9997	Newton
ME 5	30.89	0.22	0.9956	Herschel–Bulkley
ME 6	21.81	0.22	0.9654	Ostwald–de Waele
ME 7	8.390	0.50	0.9973	Ostwald–de Waele
ME 8	2.213	0.45	0.9993	Ostwald–de Waele
ME 9	0.285	1.0	0.9994	Bingham
ME 10	20.85	0.30	0.9986	Herschel–Bulkley

^1^ n represents the flow index and ^2^ R represents the correlation coefficient.

**Table 8:** Mean values of superficial parameters of the microemulsions determined at 24 ± 0.5 °C.

No.	Parameters Tested through Goniometric Technique							
	Pendant Drop (*n* = 3)		Contact Angle (*n* = 5)			W_a_ (mN/m)	W_c_ (mN/m)	S (mN/m)
	Vol (μL)	γ_LG_ (mN/m)	Vol (μL)	γ_LG_ (mN/m)	θ (°)			
1	4.96 ± 0.09	26.44 ± 0.21	5.04 ± 0.003	34.78 ± 0.525	52.14 ± 0.560	56.07 ± 0.72	69.56 ± 1.05	−13.49 ± 0.52
2	4.83 ± 0.04	25.49 ± 0.08	4.72 ± 0.003	40.85 ± 0.434	57.75 ± 0.596	62.91 ± 0.24	81.69 ± 0.86	−18.78 ± 0.77
3	4.73 ± 0.05	25.80 ± 0.03	4.85 ± 0.008	44.53 ± 1.058	48.00 ± 0.557	74.77 ± 2.23	89.05 ± 2.11	−44.52 ± 0.48
4	4.66 ± 0.03	25.38 ± 0.02	4.24 ± 0.004	35.99 ± 0.634	48.49 ± 0.438	60.56 ± 1.80	71.98 ± 1.26	−11.41 ± 1.21
5	3.51 ± 0.44	21.18 ± 0.60	3.62 ± 0.008	10.11 ± 0.095	51.10 ± 0.413	16.44 ± 0.14	20.22 ± 0.19	−3.78 ± 0.09
6	4.29 ± 0.59	23.06 ± 0.89	3.96 ± 0.003	8.05 ± 0.023	42.37 ± 0.012	13.99 ± 0.04	16.09 ± 0.04	−2.10 ± 0.007
7	3.63 ± 0.10	20.90 ± 0.49	4.25 ± 0.002	6.75 ± 0.050	47.75 ± 0.106	11.28 ± 0.08	13.50 ± 0.09	−2.21 ± 0.01
8	3.54 ± 0.23	19.08 ±0.69	4.10 ± 0.003	5.92 ± 0.036	37.03 ± 0.156	10.65 ±0.05	11.84 ± 0.07	−1.19 ± 0.02
9	5.42 ± 0.24	25.98 ± 0.49	5.27 ± 0.022	26.48 ± 1.790	53.72 ± 0.309	42.23 ± 2.96	52.95 ± 3.57	−10.72 ± 0.63
10	3.50 ± 0.32	17.97 ± 0.62	2.39 ± 0.001	5.65 ± 0.014	41.87 ± 0.057	9.84 ± 0.02	11.29 ± 0.03	−1.44 ± 0.01

**Table 9:** Kinetic descriptors specific for the miconazole release from microemulsions following the Higuchi model.

ME	1	2	3	4	5	6	7	8	9	10	Gel (R)
**D·10^−5^ (cm^2^/s)**	2.569	2.22	1.85	2.11	0.252	0.516	0.265	1.00	1.27	0.343	0.0162
**R**	0.9904	0.9842	0.9630	0.9812	0.9858	0.9910	0.9959	0.9861	0.9935	0.9961	0.9758

D represents the diffusion coefficient and R represents the correlation coefficient.

**Table 10:** Independent variables presented in a relationship with the critical quality attributes with actual and predicted responses obtained from response regression study for the analyzed microemulsions.

Variable	Independent Variables			Dependent Variables						
	Oleic Acid (%)	Tween 20 (%)	PEG 400 (%)	^1^ Ds (Actual) (nm)	Ds (Predicted) (nm)	^2^ W (Actual) (mN/m)	W (Predicted) (mN/m)	^3^ D (Actual) (cm^2^/s)	^4^ Sqrt(D) (Actual) (cm^2^/s)	Sqrt(D) (Predicted) (cm^2^/s)
Code	X_1_	X_2_	X_3_	Y_1 actual_	Y_1 predicted_	Y_2 actual_	Y_2 predicted_	Y_3 actual_	Y_3 actual_	Y_3 predicted_
ME 1	5	30	10	152.89	156.78	69.56	69.19	2.56·10^−5^	0.0051	0.0051
ME 2	5	40	10	128.90	131.04	81.69	83.79	2.22·10^−5^	0.0047	0.0047
ME 3	5	30	20	188.33	181.39	89.05	88.30	1.85·10^−5^	0.0043	0.0043
ME 4	5	40	20	119.60	125.34	71.98	73.70	2.11·10^−5^	0.0046	0.0046
ME 5	10	30	10	161.34	153.19	20.22	17.61	2.52·10^−6^	0.0016	0.0016
ME 6	10	40	10	202.29	209.24	16.09	16.38	5.16·10^−6^	0.0023	0.0023
ME 7	10	30	20	225.13	222.99	13.50	11.86	2.65·10^−6^	0.0016	0.0017
ME 8	10	40	20	250.20	248.72	11.84	13.10	1.00·10^−5^	0.0032	0.0031
ME 9	6.25	35	10	133.57	123.92	52.95	47.56	1.27·10^−5^	0.0036	0.0036
ME 10	8.75	30	15	144.36	154.01	11.29	16.68	3.43·10^−6^	0.0019	0.0018

^1^ Ds represents the mean droplet size, ^2^ W—mean work of adhesion, ^3^ D—diffusion coefficient, and ^4^ Sqrt(D)—diffusion coefficient obtained by applying a square root transformation.

**Table 11:** Analysis of Variance results for Y_1_ response.

Source	Sum of Squares	df	Mean Square	F-Value	*p*-Value
Block	2511.59	1	2511.59		
Model	14,506.93	5	2901.39	21.29	0.0150
X_1_-Oleic acid	7585.15	1	7585.15	55.67	0.0050
X_3_-PEG 400	2171.31	1	2171.31	25.94	0.0282
X_1_X_2_	3390.09	1	3390.09	24.88	0.0155
X_1_X_3_	1035.22	1	1035.22	7.60	0.0704
X_2_X_3_	459.35	1	459.35	3.37	0.1637
Residual	408.77	3	136.26		
Cor Total	17,427.29	9			

**Table 12:** Analysis of Variance results for Y_2_ response.

Source	Sum of Squares	Df	Mean Square	F-Value	*p*-Value
Block	342.05	1	342.05		
Model	8925.50	4	2231.38	115.37	0.0002
X_1_-Oleic acid	8697.67	1	8697.67	449.69	<0.0001
X_1_X_3_	41.34	1	41.34	2.14	0.2176
X_2_X_3_	89.31	1	89.31	4.62	0.0981
X_1_X_2_X_3_	125.37	1	125.37	6.48	0.0636
Residual	77.37	4	19.34		
Cor Total	9344.92	9			

**Table 13:** Analysis of Variance results for Y_3_ response.

Source	Sum of Squares	Df	Mean Square	F-Value	*p*-Value
Block	7.987·10^−7^	1	7.987·10^−7^		
Model	0.0000	6	2655·10^−6^	2361.10	0.0004
X_1_-Oleic acid	0.0000	1	0.0000	11,725.47	<0.0001
X_2_-Tween 20	6.020·10^−7^	1	6.020·10^−7^	535.41	0.0019
X_1_X_2_	6.471·10^−7^	1	6.471·10^−7^	575.58	0.0017
X_1_X_3_	4.076·10^−7^	1	4.076·10^−7^	362.55	0.0027
X_2_X_3_	2.777·10^−7^	1	2.777·10^−7^	247.01	0.0040
Residual	2.249·10^−9^	2	1.124·10^−9^		
Cor Total	0.0000	9			

**Table 14:** Setting parameters according to the findings of the factorial design to implement predictive optimization.

No.	Variables	Trial 1		Trial 2	
		Goal	Limits	Goal	Limits
1	Oleic acid	7 %	5.0–10%	in range	5–10%
2	Tween 20	maximize	30–40%	in range	30–40%
3	PEG 400	in range	10–20%	in range	10–20%
4	R_1_ (nm)	minimize	119.6–250 nm	minimize	119.6–250 nm
5	R_2_ (mN/m)	60 mN/m	11.29–89.05 mN/m	60 mN/m	11.29–89.05 mN/m
6	R_3_ (cm^2^/s)	in range	10^−5^–2.56·10^−5^ cm^2^/s	in range	10^−5^–2.56·10^−5^ cm^2^/s

## Data Availability

The data presented in this study are available in the article.
